# XBP1 links the 12-hour clock to NAFLD and regulation of membrane fluidity and lipid homeostasis

**DOI:** 10.1038/s41467-020-20028-z

**Published:** 2020-12-04

**Authors:** Huan Meng, Naomi M. Gonzales, David M. Lonard, Nagireddy Putluri, Bokai Zhu, Clifford C. Dacso, Brian York, Bert W. O’Malley

**Affiliations:** 1 Department of Molecular and Cellular Biology, Baylor College of Medicine, Houston, TX 77030 USA; 2 Dan L. Duncan Cancer Center, Baylor College of Medicine, Houston, TX 77030 USA; 3 Department of Medicine, Baylor College of Medicine, Houston, TX 77030 USA; 4 Present Address: Aging Institute of UPMC, University of Pittsburgh School of Medicine, Pittsburgh, PA 15219 USA

**Keywords:** Transcription, Transcriptional regulatory elements, Transcriptomics, Metabolism

## Abstract

A distinct 12-hour clock exists in addition to the 24-hour circadian clock to coordinate metabolic and stress rhythms. Here, we show that liver-specific ablation of X-box binding protein 1 (XBP1) disrupts the hepatic 12-hour clock and promotes spontaneous non-alcoholic fatty liver disease (NAFLD). We show that hepatic XBP1 predominantly regulates the 12-hour rhythmicity of gene transcription in the mouse liver and demonstrate that perturbation of the 12-hour clock, but not the core circadian clock, is associated with the onset and progression of this NAFLD phenotype. Mechanistically, we provide evidence that the spliced form of XBP1 (XBP1s) binds to the hepatic 12-hour cistrome to directly regulate the 12-hour clock, with a periodicity paralleling the harmonic activation of the 12-hour oscillatory transcription of many rate-limiting metabolic genes known to have perturbations in human metabolic disease. Functionally, we show that *Xbp1* ablation significantly reduces cellular membrane fluidity and impairs lipid homeostasis via rate-limiting metabolic processes in fatty acid monounsaturated and phospholipid remodeling pathways. These findings reveal that genetic disruption of the hepatic 12-hour clock links to the onset and progression of NAFLD development via transcriptional regulator XBP1, and demonstrate a role for XBP1 and the 12-hour clock in the modulation of phospholipid composition and the maintenance of lipid homeostasis.

## Abstract (web summary)

Hepatocyte 12-hour rhythms have a role in cellular stress and metabolic functions. Here, the authors demonstrate disrupting the 12-hour clock through deletion of XBP1 is associated with the development of NAFLD as well as disruption of phospholipid composition and the maintenance of lipid homeostasis.

## Introduction

Living organisms on earth, from the earliest emergence of life in the sea to the present, have evolved and sustained biological rhythms to anticipate changes in the external environment for survival. Besides the well-characterized 24-h circadian clock, 12-h periodic rhythmicity also has been noted in many intertidal plants and animals, as well as in a cluster of endoplasmic reticulum (ER) and metabolic stress genes in several peripheral mouse tissues^1–4^. Humans display multiple prominent 12-h oscillations that influence daily food intake, immune function, body temperature, cognitive performance, and various circulating hormones^5–17^. Many of these 12-h physiological cycles have connections to ER stress and unfolded protein response (UPR) pathways that intimately link to the systemic maintenance of metabolic homeostasis^18–24^. Notably, hepatic dysregulation of glucose and lipid homeostasis are hallmarks of human non-alcoholic fatty liver disease (NAFLD)^25,26^, and here, we show that hepatic ablation of *Xbp1* disrupts the 12-h clock, negatively impacts indices of metabolic fitness, and promotes a defined NAFLD phenotype.

With 12-h periodic rhythmicity, X-box binding protein 1 (XBP1s) has been shown to regulate many ER homeostatic and rate-limiting metabolic genes that control glycolysis, fatty acid oxidation, and oxidative phosphorylation^1,3,4^. Both the mRNA and protein levels of XBP1s display robust 12-h oscillations in the mouse liver that are cell-autonomous and independent of the master circadian clock regulator BMAL1 or external circadian cues^2–4^. The recent discovery of 12-h rhythmicity in both global gene expression and rhythmic activation of the ER stress transcriptional factor XBP1 in the mouse liver offers unique opportunities to define the role of 12-h clock oscillators that underlie normal physiology and metabolic disease. Here, we have extended our characterization of XBP1 as a molecular component of the 12-h clock^4^, showing that XBP1 plays a key role in controlling membrane fluidity and its ability to properly maintain phospholipid composition and lipid homeostasis, linking the 12-h clock to the onset and progression of a type of NAFLD development via XBP1s transcriptional regulation. These findings suggest that the XBP1-dependent 12-h clock is important for the liver to maintain healthy metabolic homeostasis.

## Results

### Hepatic disruption of the 12-h clock is associated with progressive NAFLD

**Fig. 1:**
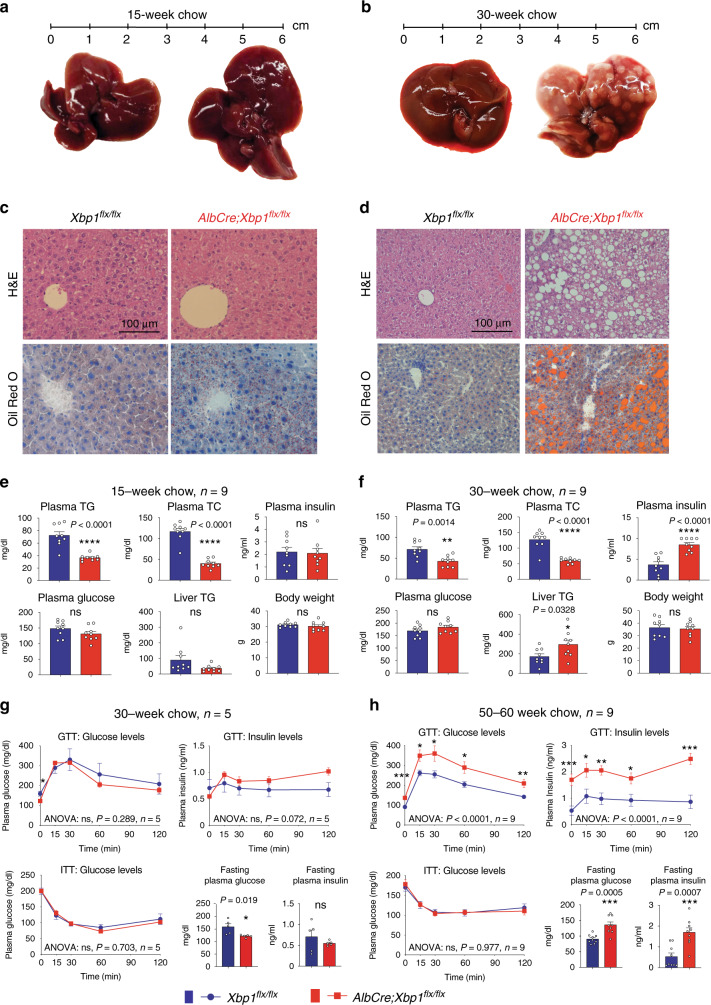
Liver-specific *Xbp1* ablation links hepatic disruption of the 12-h clock to progressive NAFLD. **a**–**d** Liver gross morphology, histology and liver functional parameters of the *Xbp1*^*flx/flx*^ and *AlbCre;Xbp1*^*flx/flx*^ mice. **a**, **b** Liver gross morphology of the *Xbp1*^*flx/flx*^ and *AlbCre;Xbp1*^*flx/flx*^ mice fed regular chow ad libitum for 15 (**a**) or 30 (**b**) weeks age. **c**, **d** Hematoxylin and eosin (H&E) and Oil Red O (ORO) staining of liver sections from the indicated mouse strains for 15 (**c**) or 30 (**d**) weeks age. Scale bar, 100 μm. H&E shows tissue composition, macrovesicular fat, and hepatocyte ballooning in the livers of *AlbCre;Xbp1*^*flx/flx*^ mice. ORO visualizes neutral lipid droplets. **e**, **f** Plasma triglycerides (TG), plasma total cholesterol (TC), plasma insulin, plasma glucose, liver TG, and body weight in the indicated mouse strains (*n* = 9) for 15 (**e**) or 30 (**f**) weeks age. **g**, **h** Glucose tolerance test (GTT), insulin tolerance test (ITT), and fasting plasma glucose and insulin levels from the indicated mouse strains for 30 (**g**) *n* = 5 or 50–60 (**h**) *n* = 9 weeks age. Unpaired Student’s *t*-test and one-way ANOVA analysis were performed with *p* value indicated. Data are graphed as the mean ± SEM. *p < 0.05, **p < 0.005, ***p < 0.001, ****p < 0.0001.

In the present study, we set out to identify and characterize the physiological consequences of 12-h clock dysregulation by liver-specific ablation of *Xbp1*, which we found to be a transcriptional regulator of 12-h gene oscillations^4^. We generated liver-specific *Xbp1* knockout mice (*AlbCre;Xbp1*^*flx/flx*^) using the promoter of the serum albumin gene to drive expression of *Cre* recombinase (*AlbCre*) in *Xbp1* floxed mice with loxP sites flanking exon 2 of the *Xbp1* gene (*Xbp1*^*flx/flx*^) in the C57BL/6J background and examined *Xbp1* expression to confirm its ablation specifically in the mouse liver (Supplementary Fig. 1a, b). To determine whether molecular disruption of the 12-h clock via *Xbp1* ablation corresponds with chronic abnormalities in liver biology and metabolic functions, we analyzed the *AlbCre;Xbp1*^*flx/flx*^ mice in an age-dependent manner. Consistent with previous reports^27,28^, the *AlbCre;Xbp1*^*flx/flx*^ mice displayed hypocholesterolemia and hypotriglyceridemia in the plasma without any significant liver histological, functional or gross phenotypic abnormalities throughout the first 15 weeks of life (Fig. 1a–c), suggesting that liver-specific ablation of *Xbp1* did not noticeably affect fetal growth or liver development. We noted that previous studies focusing on spontaneous XBP1 function were conducted in young mice at 3–4 months of age, and none of these earlier animal studies monitoring hepatic steatosis were aware of the robust 12-h clock rhythmicity that exists in the ER stress and UPR pathways^27,28^.

Notably, although the *AlbCre;Xbp1*^*flx/flx*^ mice did not show any noticeable gross liver morphology during the first 15 weeks, we found that these mice fed regular chow ad libitum had markedly elevated liver steatosis by 20 weeks of age, with all the *AlbCre;Xbp1*^*flx/flx*^ mice exhibiting severe fatty liver as evidenced by fat deposition throughout all liver lobes by 30 weeks of age (Fig. 1b, d). Further histological analysis at this stage revealed abnormal accumulation of hepatic lipid droplets, macrovesicular fat, and hepatocyte ballooning, along with a significant increase in liver triglyceride (TG) levels (Fig. 1e, f); this phenotype was not accompanied by any apoptotic or inflammatory features, nor by liver functional damage (Supplementary Fig. 2a, b). The *AlbCre;Xbp1*^*flx/flx*^ mice also displayed hyperinsulinemia without significant changes in glucose levels and body weight by 30 weeks of age (Fig. 1f). To further determine the impact of chronic 12-h clock disruption on glucose metabolism and homeostasis, we examined the *AlbCre;Xbp1*^*flx/flx*^ mice fed regular chow *ad libitum* by 30 weeks and 50–60 weeks of age using glucose tolerance test (GTT) and insulin tolerance test (ITT). We found that the *AlbCre;Xbp1*^*flx/flx*^ mice displayed significantly impaired fasting glycemia, hyperinsulinemia, and impaired glucose tolerance by 50–60 weeks of age (Fig. 1g, h), suggesting that hepatic loss of *Xbp1* led to a state of metabolic dysregulation similar to that observed in human NAFLD. GTTs further revealed a significant elevation in insulin release in response to the increased glucose levels in 50–60 week *AlbCre;Xbp1*^*flx/flx*^ mice, while these animals displayed normal insulin tolerance (Fig. 1h), suggesting that the progressively impaired glucose tolerance upon liver-specific ablation of *Xbp1* unlikely involves a primary defect in pancreatic function or insulin resistance. Collectively, these results demonstrate that hepatic ablation of *Xbp1* in the *AlbCre;Xbp1*^*flx/flx*^ mouse model disrupts the 12-h clock and promotes the onset and progression of a type of spontaneous NAFLD development.

### 12-h clock perturbation links to NAFLD phenotype

The defined NAFLD phenotype manifested from ablation of 12-h clock regulator *Xbp1* can be promoted by the consequences of direct 12-h clock dysfunction, or it could arise through indirect perturbation of core circadian clock components^3,4^. To distinguish between these two possibilities, we profiled the temporal characteristics of oscillating transcriptomes from adult littermates at 12–16 weeks of age, wherein the *AlbCre;Xbp1*^*flx/flx*^ mice did not yet manifest any noticeable NAFLD phenotype promoted by *Xbp1* ablation. We entrained the *Xbp1*^*flx/flx*^ and *AlbCre;Xbp1*^*flx/flx*^ mice to a 12-h light:12-h dark cycle (12 h:12 h LD) under constant temperature for two weeks, and then transferred the mice to constant darkness (see “Methods”). To determine whether direct perturbation of the 12-h clock via *Xbp1* ablation promotes the consequent NAFLD phenotype, we sampled two biological replicates of mouse liver tissues at a high temporal resolution starting at circadian time 0 (CT0) and proceeding every two hours for four complete 12-h cycles (48 h in total, two complete circadian cycles), and used RNA-Sequencing (RNA-Seq) to profile their liver transcriptomes (Supplementary Figs. 3, 4, and Supplementary Data 1 and Supplementary Data 2).

**Fig. 2:**
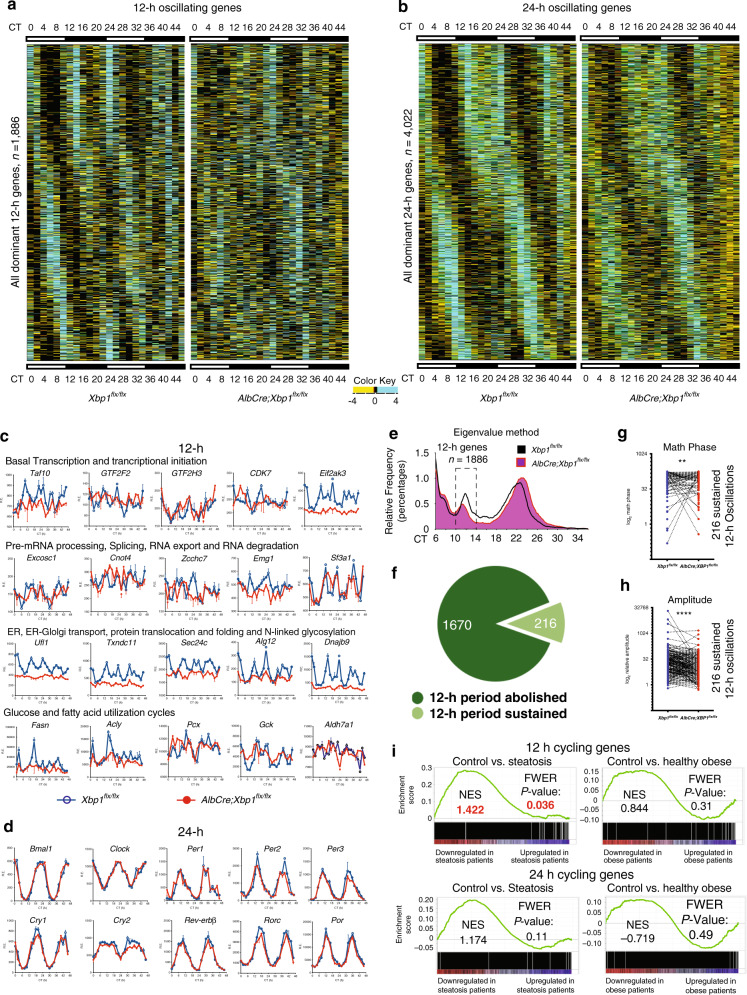
Perturbation of the 12-h clock, but not the core circadian clock, is associated with the onset and progression of the NAFLD phenotype. Rhythmic transcripts detected by RNA-Seq and the eigenvalue/pencil method were plotted as heatmaps for 12-h and 24-h cycling genes. **a** Heatmaps of the 12-h oscillating transcripts for the *Xbp1*^*flx/flx*^ and *AlbCre;Xbp1*^*flx/flx*^ mice (*n* = 1886). **b** Heatmaps of the 24-h oscillating transcripts (*n* = 4022). **c** Representative 12-h gene oscillations plotted against circadian time (CT) from 2-h resolution transcriptome profiling of the *Xbp1*^*flx/flx*^ (blue lines) and *AlbCre;Xbp1*^*flx/flx*^ (red lines) mice. **d** Representative 24-h gene oscillations of core components of the circadian clock plotted against CT time from 2-h resolution transcriptome profiling of the *Xbp1*^*flx/flx*^ and *AlbCre;Xbp1*^*flx/flx*^ mice. **e** The distribution of periods of all dominant oscillations identified by RNA-Seq and the eigenvalue/pencil approach. **f** The proportion of the 12-h gene oscillations that had abolished (*n* = 1670) or sustained (*n* = 216) 12-h periods in the *AlbCre;Xbp1*^*flx/flx*^ mice. **g**, **h** The matched-pairs of individual plots of mathematical phase (**g**) and amplitude (**h**) for the 216 sustained 12-h gene oscillations in the *Xbp1*^*flx/flx*^ and *AlbCre;Xbp1*^*flx/flx*^ mice. **i** Gene set enrichment analysis (GSEA) of the 12-h and 24-h cycling genes enriched in human hepatic steatosis (left) and healthy obese (right) gene expression datasets (GSE48452). Normalized enrichment scores (NES) and FWER *p* values are shown.

To allow unbiased characterization of all superimposed oscillations and to avoid the period pre-assignment bias of the established rhythmic algorithms such as JTK_CYCLE and ARSER^1,4^, we used the eigenvalue/pencil method to provide unbiased detection of both 12-h and 24-h oscillations in transcript abundance^4,29^. We leveraged the high temporal resolution of our RNA-Seq data to accurately identify the superimposed oscillations for all genes by the eigenvalue/pencil method. We used the computational criteria of 12-h and 24-h oscillations that have been previously set by Hughes et al.^1^; The term “12-h oscillating gene” was defined as any gene identified as cycling with a dominant 10–14-hperiod and “24-h oscillating gene” as those with a dominant 20–28-h period. In this context, we determined a total of 1886 12-h oscillating genes and 4022 24-h oscillating genes in the mouse liver (Fig. 2a–d and Supplementary Data 3). To determine whether liver-specific ablation of *Xbp1* globally impairs the hepatic 12-h transcriptome, we then examined the period, phase and amplitude of all 1886 12-h oscillating genes in the *AlbCre;Xbp1*^*flx/flx*^ mouse liver (Fig. 2e–h).

We found that 1670 of 1886 (88.55%) 12-h oscillating genes no longer possessed a 12-h periodicity in the *AlbCre;Xbp1*^*flx/flx*^ mice (Fig. 2f), and the remaining 216 (11.45%) 12-h oscillating genes showed disrupted amplitude or phase in the *AlbCre;Xbp1*^*flx/flx*^ mice (Fig. 2f–h), indicating that XBP1 globally regulates the hepatic 12-h transcriptome in vivo. To make certain of the robustness of these eigenvalue/pencil findings, we also performed an additional statistical analysis of the primary data using the independent rhythmicity analysis incorporating non-parametric (RAIN) method^30^. RAIN analysis of the primary data detected 2396 and 4772 12-h oscillating genes at *p* < 0.005 and *p* < 0.05, respectively (Supplementary Fig. 5a and Supplementary Data 4). Of the 1886 dominant 12-h oscillating genes that were identified by our eigenvalue/pencil method, 1213 (64.42%) 12-h genes also were detected by the independent RAIN method, strongly suggesting the prevalence and robustness of our characterization of the hepatic 12-h oscillations. Consistent with our major conclusions via eigenvalue/pencil analysis, at RAIN *p* < 0.005 for example, 1827 of 2396 (76.25%) 12-h oscillating genes no longer possessed a 12-h periodicity and the remaining 569 (23.75%) 12-h oscillating genes showed disrupted phase or peak shape in the *AlbCre;Xbp1*^*flx/flx*^ mice (Supplementary Fig. 5b), strongly supporting our major conclusions that XBP1 globally regulates the hepatic 12-h transcriptome in vivo.

By contrast, all core components of the circadian clock remained unaffected (Fig. 2b, d), suggesting that the disrupted 12-h gene oscillations are the direct consequence of hepatic *Xbp1* ablation and are not driven by secondary perturbations of the circadian clock. Consistently, by gene set enrichment analysis (GSEA) of 12-h gene oscillations against the gene expression profiles of human NAFLD patients that have been previously published^31^, we found that human NAFLD progression, but not healthy steatosis, is significantly associated with the 12-h transcriptome (Fig. 2i and Supplementary Fig. 6a, b); this strongly suggests that the 12-h clock could play an important role in the regulation of metabolic homeostasis and its disruption could lead to NAFLD development. Collectively, these observations demonstrate that direct perturbation of the 12-h clock, but not the core circadian clock, is associated with this spontaneous NAFLD phenotype.

### XBP1s regulates the hepatic 12-h cistrome

**Fig. 3:**
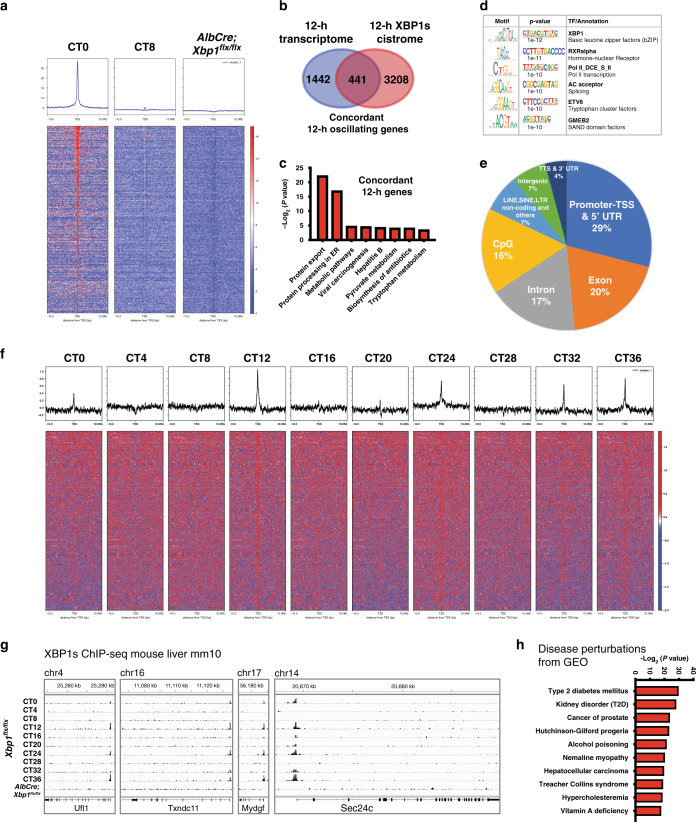
XBP1s regulates the hepatic 12-h cistrome. **a** Binding profiles and heatmaps of XBP1s at the transcription start sites (TSSs) of 441 concordant 12-h oscillating genes in *Xbp1*^*flx/flx*^ at CT0 (left) and CT8 (middle), and in the *AlbCre;Xbp1*^*flx/flx*^ mice (right). The *y* axis represents the average signal in a 50-bp bin (normalized uniquely mapped reads). **b** Venn diagram of the 12-h transcriptome (*n* = 1886) and the 12-h XBP1s cistrome (*n* = 3649). Intersection: concordant 12-h oscillating genes (*n* = 441). **c** Top-enriched KEGG pathways identified in the concordant 12-h oscillating genes. **d** Top-enriched transcriptional factor motifs identified from concordant 12-h oscillating genes. **e** The proportion of XBP1s cistrome in the concordant 12-h oscillating genes. **f** 12-h rhythmic XBP1s cistrome binding detected by ChIP-Seq were plotted as binding profiles and heatmaps for the concordant 12-h oscillating genes every 4 CT hours for 36 h in total. **g** Representative views of 12-h rhythmic XBP1s ChIP-Seq data at the *Ufl1*, *Txndc11*, *Mydgf*, and *Sec24c* gene loci. **h** Top-enriched disease perturbations identified from concordant 12-h oscillating genes.

To further define the XBP1-dependent 12-h clock cistrome in the liver, we used chromatin immunoprecipitation sequencing (ChIP-Seq) to identify the in vivo DNA binding sites for XBP1s, the active spliced form of XBP1, in the livers of age-matched 12–16-weeks-old mice. Based on the temporal characteristics of the 12-h cycling rhythmic transcriptomes enriched at dawn (CT0) and dusk (CT12), we chose liver samples at CT0 and CT8 (four hours before the dusk 12-h rhythmic peak) to interrogate the genomic binding profiles of XBP1s in vivo (Fig. 3a and Supplementary Data 1). We identified a total of 4760 high-confidence XBP1s binding sites at 3649 coding genes, 441 of which overlap with XBP1-dependent 12-h oscillating genes that were identified in the hepatic 12-h transcriptome (Fig. 3b); herein we defined them as “concordant 12-h oscillating genes”. Consistently, the concordant 12-h oscillating genes were enriched in KEGG pathways for protein export, ER protein processing, and a number of metabolic pathways (Fig. 3c and Supplementary Fig. 7a, b), indicating a significant involvement of concordant 12-h rhythms in coordinating oscillations of ER stress and metabolic processing to ensure systemic energy homeostasis.

ChIP-Seq motif analysis confirmed that the hepatic XBP1s binding sites at the concordant 12-h oscillating genes were strongly associated with bZIP leucine zipper containing transcription factors (TFs) binding sites (Fig. 3d), which contain the known XBP1 consensus DNA binding motif^32^. In addition, we identified a number of enriched motifs from several TFs, including retinoid X receptor alpha (RXRα), ETS variant 6 (ETV6), and glucocorticoid modulatory element-binding protein 2 (GMEB2) (Fig. 3d), which are known to play key roles in signaling activity in development and in transcriptional dysregulation in cancer^33–35^, suggesting that additional nuclear TFs also may participate in the co-regulation of the XBP1 concordant 12-h oscillating transcriptome. A subsequent detailed annotation revealed that the hepatic XBP1s binding sites were preferentially enriched at proximal promoter regions (29%) associated with transcriptional initiation, which is consistent with the known human XBP1s cistrome distribution^32^, with additional enrichment binding at intron (17%) and exon (20%) regions respectively (Fig. 3e); this suggests that transcriptional elongation, nascent RNA processing and/or splicing in addition to transcriptional initiation also may play an important role in the regulation of 12-h oscillating gene expression. Interestingly, we identified substantial CpG, LINE, SINE, and LTR binding enrichment at intergenic regions (Fig. 3e), suggesting that additional epigenetic mechanisms also may contribute to the 12-h clock regulation.

To further determine whether XBP1s regulates the hepatic 12-h clock cistrome under physiological conditions, we performed XBP1s ChIP-Seq in mouse liver tissues starting from CT0 and proceeding every four hours for three complete 12-h cycles (36 h in total) and revealed that hepatic XBP1s directly regulates concordant 12-h oscillating gene expression in a 12-h rhythmic manner (Fig. 3f, g). Notably, the concordant 12-h oscillating genes were strongly associated with human disease perturbations connected to type 2 diabetes mellitus, prostate cancer, hepatocellular carcinoma, and several genetic diseases associated with tissue development and metabolic disorders (Fig. 3h).

### *Xbp1* ablation causes lipidomic impairment in PC-LPC cycle

**Fig. 4:**
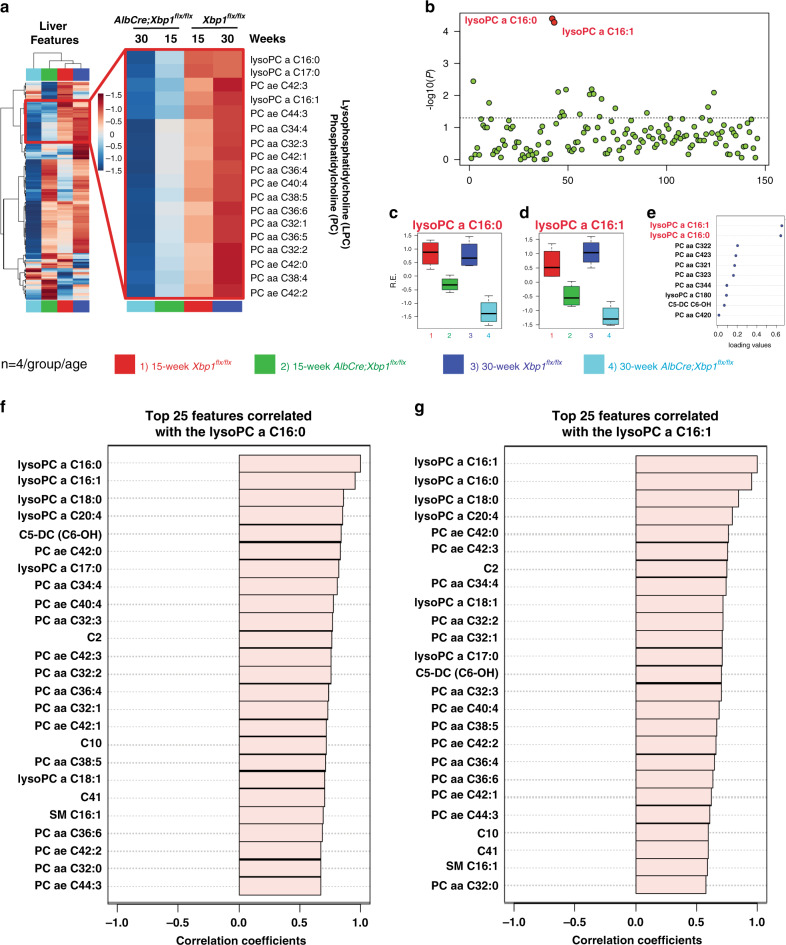
Ablation of hepatic *Xbp1* causes a lipidome-wide impairment in the phosphatidylcholine (PC)-lysophosphatidylcholine (LPC) cycle pathway. **a** The overall heatmap of the essential metabolites and lipid species in the indicated mouse livers at 15- and 30 weeks of age by the standard AbsoluteIDQ p180-based targeted metabolomics of whole liver extracts (left panel). The indicated PC and LPC cycle lipid species (right panel) were identified as the top impaired cluster in the *AlbCre;Xbp1*^*flx/flx*^ mouse livers (*n* = 4 per age/group). **b**–**e** Univariate analysis identified lysoPC a C16:0 and lysoPC a C16:1 as the two most important features in the *AlbCre;Xbp1*^*flx/flx*^ mouse livers of the two indicated age groups (*n* = 4 per age/group). One-way ANOVA was performed in (**b**) to tell the important features with *p* value threshold 0.05 indicated as the dotted horizontal line, followed by post hoc Fisher’s least significant difference method (Fisher’s LSD). Individual box plots for the two indicated lysoPC features in the two age groups were shown in (**c**) and (**d**) (the *y* axis represents the average normalized levels). The bar plots show the normalized values (mean ± SEM). The boxes range from 25 and 75% percentiles; the 5 and 95% percentiles are indicated as error bars. Medians are indicated by horizontal lines within each box. Significant features identified by the discriminant analysis (sPLS-DA) are shown in (**e**) (the *x* axis represents the absolute loading values of the ranking features). **f**, **g** Top metabolites and lipid species correlated with the two most important features in the *AlbCre;Xbp1*^*flx/flx*^ mouse livers, lysoPC a C16:0 (**f**), and lysoPC a C16:1 (**g**).

One major mechanism of 12-h clock regulation involves the cycling of genes that have important roles in the maintenance of hepatic metabolism (Figs. 2c, 3c). Thus, we examined key metabolites and lipid species in mouse livers at 15 weeks and 30 weeks of age by targeted metabolomics from major metabolite and lipid classes including acylcarnitines, phosphatidylcholine (PC), lysophosphatidylcholines (LPC), and sphingomyelins (See metabolomics profiling in “Methods” and Supplementary Data 5). Surprisingly, the *AlbCre;Xbp1*^*flx/flx*^ mouse livers revealed dramatic lipidome-wide defects in the PC-LPC cycle pathways (Fig. 4a and Supplementary Figs. 8–10). Moreover, we found that lysoPC a C16:0 and lysoPC a C16:1 were the top lipid species that were significantly reduced in the PC-LPC cycle pathway in an ‘age-dependent manner’ from the *AlbCre;Xbp1*^*flx/flx*^ mice (Fig. 4b–e), consistent with the onset and progression of the NAFLD phenotype. Further correlation analyses revealed that not only both types of the PC and lysoPC species, but also the fatty acyl composition of phospholipids such as the monounsaturated palmitoleic acid (16:1) and unsaturated arachidonic acid (20:4), were dysregulated in the *AlbCre;Xbp1*^*flx/flx*^ mouse liver (Figs. 4a, f, g), suggesting an unexpected link between the 12-h clock and multiple rate-limiting metabolic pathways via *Xbp1* ablation in fatty acid unsaturation and PC-LPC cycles.

### *Xbp1* regulates membrane fluidity and metabolic function

**Fig. 5:**
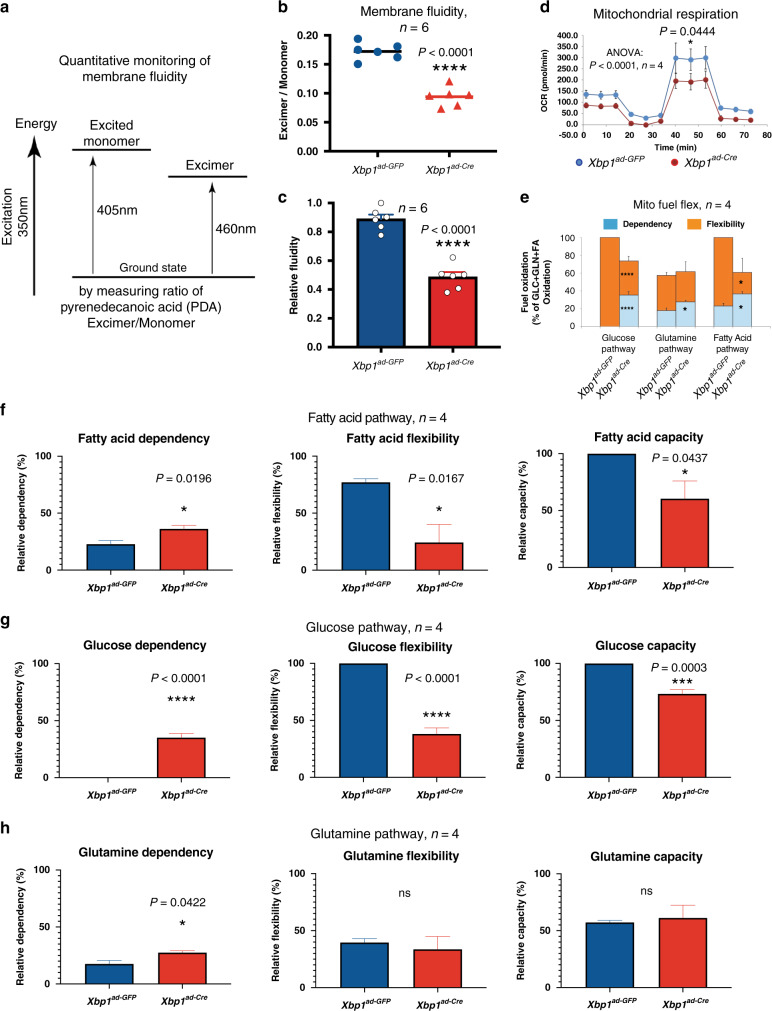
*Xbp1* ablation significantly reduces cellular membrane fluidity and impairs glucose and lipid utilization. **a**–**c** Membrane fluidity of mouse embryonic fibroblasts (MEFs) measured using a fluorescent lipophilic probe, pyrenedecanoic acid (PDA) (**a**). The absolute excimer: monomer (**b**) and relative ratio (**c**) of emission at 460 nm to emission at 405 nm are shown, *n* = 6 independent *Xbp1*^*ad-GFP*^ and *Xbp1*^*Ade-Cre*^ MEFs. **d**–**h** Seahorse metabolic profiling of the indicated MEF strains, *n* = 4 independent *Xbp1*^*ad-GFP*^ and *Xbp1*^*Ade-Cre*^ MEFs. Cell Mito Stress test profile (**d**) for fundamental mitochondrial function, and Mito Fuel Flex test profile for substrate fuel dependency, overview (**e**), and the three major fuel pathways of fatty acid (**f**), glucose (**g**), and glutamine (**h**) are shown. Unpaired Student’s *t*-test and one-way ANOVA analysis were performed with *p* value indicated. Data are graphed as the mean ± SEM. *p < 0.05, **p < 0.005, ***p < 0.001, ****p < 0.0001. OCR oxygen consumption rate, GLC glucose, GLN glutamine, FA fatty acids.

Because PC and LPC are critical lipid species for maintenance of membrane composition and structure of organelle and cellular membranes^36–38^, we next investigated whether ablation of *Xbp1* leads to alterations in membrane fluidity. Pyrenedecanoic acid (PDA) is a lipid analog probe that can be detected as a monomer by ~405 nm fluorescence when excited with 350 nm excitation^39^. Once this lipophilic pyrene probe forms a spatial interaction at cellular membranes, it undergoes excimer formation that can be detected by emission at ~460 nm (Fig. 5a)^40^. In this context, we examined the pyrene fluorescence-mediated fluidity and identified a remarkable low (rigid) membrane fluidity both in *AlbCre;Xbp1*^*flx/flx*^ primary hepatocytes and *Xbp1*^*ad-Cre*^ mouse embryonic fibroblasts (MEFs) (Fig. 5a–c, and Supplementary Fig. 11a), suggesting an unappreciated cell-autonomous link between the 12-h clock and membrane fluidity-associated processes via XBP1 regulation. The viscosity of cellular membranes is a fundamental property that limits lateral diffusion of molecules within the lipid bilayer and affects the activity of membrane-associated processes, including many rate-limiting metabolic processes in ER, mitochondria, and Golgi complexes^23,41–46^. To determine how the perturbation of XBP1-regulated membrane fluidity impacts cellular metabolic function, we examined mitochondrial functional parameters and determined that ablation of *Xbp1* compromised both mitochondrial respiration and non-mitochondrial oxygen consumption (Fig. 5d and Supplementary Fig. 11b, c). Furthermore, we performed Mito Fuel Flex analyses under nutrient deprivation and determined that *Xbp1* ablation significantly compromised the flexibility and capacity of cells to utilize glucose and fatty acid, but not glutamine, for fuel utilization (Fig. 5e–h). Collectively, these results indicate an unexpected link between 12-h clock gene expression, cellular membrane fluidity, and mitochondrial utilization of fatty acid and glucose substrates via XBP1 regulation.

### The 12-h clock regulates phospholipid remodeling pathways

**Fig. 6:**
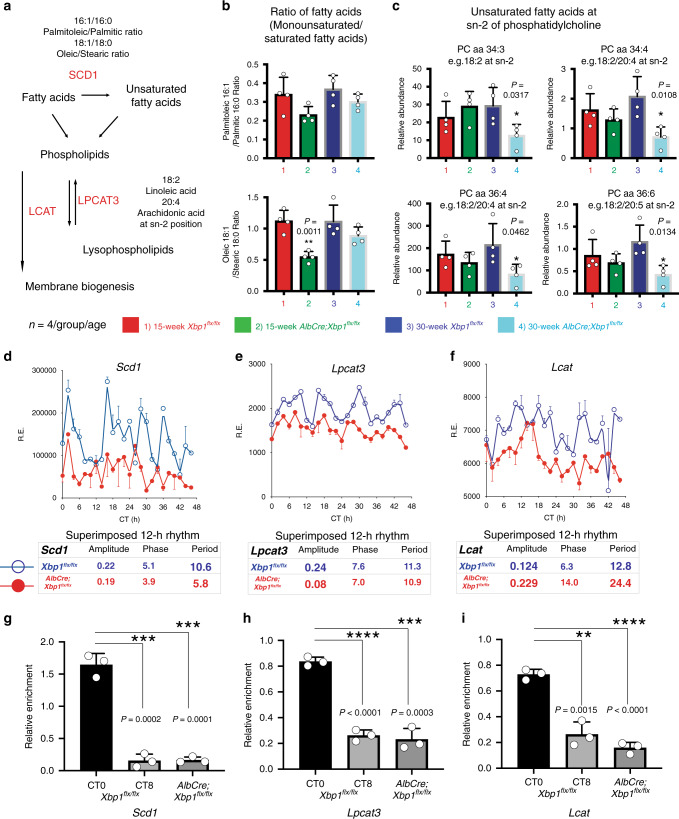
The 12-h clock links membrane fluidity to the rate-limiting metabolic processes in fatty acid monounsaturated and phospholipid remodeling pathways. **a** The schematic diagram of the fatty acid monounsaturated and phospholipid remodeling pathways. The key enzymes SCD1, LPCAT3, and LCAT are shown. **b** The ratio of palmitoleate (16:1) to palmitate (16:0) (upper panel) and oleate (18:1) to stearate (lower panel) in total liver lipid extracts are shown (*n* = 4 per group). **c** Metabolomic analysis of the relative abundance of the PC species in the indicated mouse livers at 15- and 30 weeks of age (*n* = 4 per group). **d**–**f** 12-h gene oscillations of *Scd1* (**d**), *Lpcat3* (**e**), and *Lcat* (**f**) plotted against circadian time (CT) from 2-h resolution transcriptome profiling of the *Xbp1*^*flx/flx*^ (blue lines) and *AlbCre;Xbp1*^*flx/flx*^ (red lines) mice (*n* = 2 per timepoint). The respective superimposed 12-h rhythmic eigenvalue/pencil parameters of each gene are shown in the lower panels. **g**–**i** ChIP-Seq binding enrichment of XBP1s at gene loci of *Scd1*, chr19:44,388,626-44,388,882 (**g**), *Lpcat3*, chr6:124,679,113-124,679,428 (**h**), and *Lcat*, chr8:10,594,301-105,943,367 (**i**) in *Xbp1*^*flx/flx*^ at CT0 (left) and CT8 (middle), and in the *AlbCre;Xbp1*^*flx/flx*^ mice (right) (*n* = 3 per group). The *y* axis represents the average signal in a 5-bp bin (normalized to 500 bp uniquely mapped reads). Unpaired Student’s *t*-test analysis was performed with *p* value indicated. Data are graphed as the mean ± SEM. *p < 0.05, **p < 0.005, ***p < 0.001, ****p < 0.0001.

Because the fatty acyl composition of phospholipids determines the biophysical properties of membranes including membrane fluidity^47,48^, we further characterized the changes in the saturated and unsaturated modifications in fatty acyl composition in mouse livers at 15 weeks and 30 weeks of age. Mammalian saturated fatty acids possess a diverse and asymmetric distribution that is primarily formatted by a phospholipid remodeling pathway called the Lands cycle^49^. Accordingly, while lecithin–cholesterol acyltransferase (LCAT, also called phosphatidylcholine–sterol O-acyltransferase) converts PCs to LPCs when a fatty acid is removed from plasma PC and transferred to cholesterol^50^, lysophosphatidylcholine acyltransferase 3 (LPCAT3) preferentially catalyzes the formation of PCs from LPCs containing unsaturated fatty acids in the liver, especially linoleic acid (18:2) and arachidonic acid (20:4) at the *sn-2* position (Fig. 6a)^51–53^. In addition, stearoyl-CoA desaturase 1 (SCD1) is the key enzyme that synthesizes the monounsaturated fatty acids, palmitoleic acid (16:1) and oleic acid (18:1), from the Δ9-desaturation of the saturated fatty acids, palmitic acid (16:0) and stearic acid (18:0), respectively (Fig. 6a)^54^. In this context, we examined the ratios of fatty acids and the abundance of unsaturated fatty acids at *sn-2* of PC species and found that the *AlbCre;Xbp1*^*flx/flx*^ mouse livers had significant defects in both the fatty acid monounsaturated and phospholipid remodeling pathways (Fig. 6b, c). Specifically, we observed that the ratios of oleic and stearic acids were significantly reduced at 15-weeks of age (Fig. 6b), and the PC species containing unsaturated fatty acids, including linoleic and arachidonic acids at the *sn-2* position, were markedly decreased at 30 weeks of age in the *AlbCre;Xbp1*^*flx/flx*^ mouse livers (Fig. 6c).

Given the enzymatic activity of SCD1, LPCAT3, and LCAT, we reasoned that the XBP1-dependent 12-h clock might have a role in the regulation of the fatty acid monounsaturated and phospholipid remodeling pathways. Indeed, we found that the hepatic mRNA levels of both *Scd1, Lpcat3*, and *Lcat* genes possess robust 12-h oscillations, whereas *Scd1* and *Lcat* no longer possessed 12-h period, while *Lpcat3* showed a disrupted amplitude in the *AlbCre;Xbp1*^*flx/flx*^ mouse liver (Fig. 6d–f). Consistently, temporal XBP1s occupancy at the respective *Scd1, Lpcat3*, and *Lcat* gene loci showed concomitant rhythmic enrichment at CT0, but not CT8, in the mouse liver (Fig. 6g–i), indicating that hepatic XBP1s directly regulates the temporal 12-h transcription of *Scd1*, *Lpcat3*, and *Lcat* in vivo. Collectively, these data suggest that the 12-h clock directly links membrane fluidity to rate-limiting metabolic processes in fatty acid monounsaturated and phospholipid remodeling pathways, at least partially through mechanisms associated with XBP1 temporal transcriptional regulation of the key metabolic enzymes such as SCD1, LPCAT3, and LCAT.

### Ablation of XBP1 abolishes 12-h RER oscillation

**Fig. 7:**
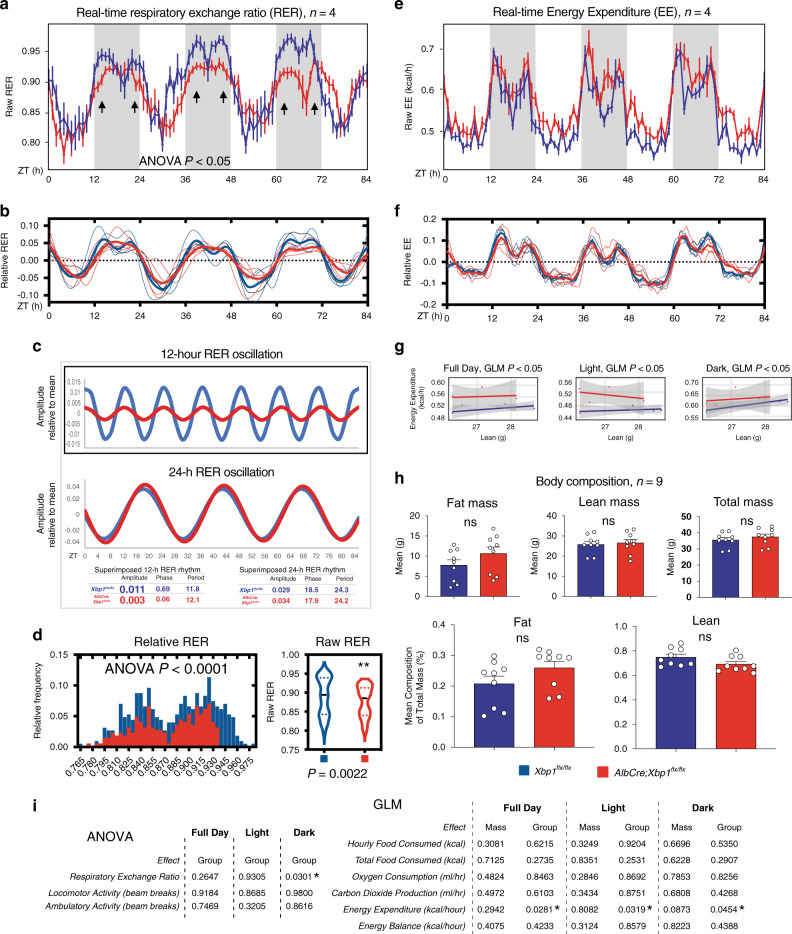
Disruption of the liver-specific 12-h clock abolishes 12-h oscillation of respiratory exchange ratio (RER). **a**, **b** The raw (**a**) and relative (**b**) real-time RER values of the indicated mice (*n* = 4) housed under ad libitum feeding conditions. Individual (light line) and averaged (bold line) real-time RER values of the indicated mouse strains plotted against the indicated time points. **c** Eigenvalue/pencil decompositions and deconvolutions of the mean RER oscillations of 12-h (upper panel) and 24-h (lower panel) oscillations**. d** The relative frequency histogram (left panel) and raw values (right panel) of real-time RER of the indicated mice housed under ad libitum feeding conditions (*n* = 4). **e**, **f** The raw (**e**) and relative (**f**) real-time energy expenditure (EE) values of the indicated mice (*n* = 4) housed under ad libitum feeding conditions. Individual (light line) and averaged (bold line) real-time EE values of the indicated mouse strains plotted against the indicated time points. **g** The ANCOVA analysis (generalized linear model, GLM) of the real-time EE in (**e**, **f**). normalized by lean mass at the indicated light, dark, and full-day conditions (*n* = 4). **h** The body composition of the indicated mice (*n* = 9) housed under ad libitum feeding conditions. Unpaired Student’s *t*-test and one-way ANOVA analysis were performed with *p* value indicated. **i** The integrated statistical analysis is shown for the indirect calorimetry experiments of the *Xbp1*^*flx/flx*^ and *AlbCre;Xbp1*^*flx/flx*^ mice (*n* = 4) housed under ad libitum feeding conditions. One-way ANOVA analysis with Tukey’s post hoc analysis was performed with *p* value threshold 0.05. Blue line*: Xbp1* ^*flx/flx*^; red line: *AlbCre;Xbp1* ^*flx/flx*^. Mouse RER and EE data are graphed as the mean ± SEM. *p < 0.05, **p < 0.005, ***p < 0.001, ****p < 0.0001.

Because low membrane fluidity also affects a variety of cellular processes of the plasma membrane by dysregulating the transportation of biological materials, including different types of lipoproteins that are important for the maintenance of the systemic homeostasis^36–38,55^, we speculated that the hepatic disruption of the 12-h clock via XBP1 might promote a chronic defect in whole-body energetics in addition to the compromised intracellular mitochondrial activity. Indeed, previous studies have demonstrated that ablation of *Xbp1* in the liver resulted in a profound reduction of TG transport upon selective inhibition of the breakdown of very-low-density lipoprotein (VLDL)^27,28^, which carries TGs together with cholesterol to peripheral tissues^56,57^. To determine the impact of metabolic dysregulation of the hepatic 12-h clock on the synchronization of systemic energetics, we performed metabolic profiling of mice fed ‘regular chow’ ad libitum at 50 weeks of age. By measuring the real-time respiratory exchange ratio (RER), we identified a disruption in RER in the *AlbCre;Xbp1*^*flx/flx*^ mice (Fig. 7a–d), and this phenotype is remarkable in that it is accompanied by a rhythmic disruption in RER without any noticeable changes in feeding behavior or activity (Fig. 7a, b and Supplementary Fig. 12a, b). Thus, we applied the unbiased eigenvalue/pencil method to the RER metabolic profiles and observed that the superimposed 12-h rhythmicity was specifically abolished in the *AlbCre;Xbp1*^*flx/flx*^ mice, whereas the 24-h rhythmicity remained unaffected (Fig. 7c). Furthermore, we performed a complete statistical analysis of the energy expenditure (EE) and whole-body composition by CalR^58^, an analytical tool integrated with the general linear model (which includes ANOVA and ANCOVA), allowing for greater flexibility in the interpretation of the energetic balance^58,59^. We found a statistically significant decrease (by ANOVA) in the 12-h RER and a modest increase (by ANCOVA, generalized linear model, GLM) in EE in the *AlbCre;Xbp1*^*flx/flx*^ mice (Fig. 7d–g and Supplementary Data 6), whereas other factors including body composition (a modest increase in fat composition but no statistical significance), oxygen consumption, carbon dioxide production, and locomotor and ambulatory activities had no statistical or rhythmic changes in the *AlbCre;Xbp1*^*flx/flx*^ mice (Fig. 7h, i and Supplementary Fig. 12c–f). These data revealed that chronic disruption of the liver-specific 12-h clock by *Xbp1* ablation leads to perturbations in 12-h RER oscillation that may disrupt and redistribute the systemic balance of whole-body energetics, suggesting that the 12-h clock plays an important role in coordinating hepatic 12-h oscillations with whole-body energetics.

## Discussion

Driven by the hypothesis that a molecular clock establishing 12-h periodic rhythmicity exists to coordinate ER stress and metabolic oscillations to ensure systemic energy homeostasis, we set out to determine the role of the 12-h clock in energy homeostasis. Our findings revealed a link between chronic disruption of 12-h rhythmicity via *Xbp1* ablation in mouse liver, and the onset and progression of a type of NAFLD development occurring under ‘normal chow feeding’. We show that the ablation of *Xbp1* disrupts the 12-h clock and compromises mitochondrial respiration and non-mitochondrial oxygen consumption, reducing the flexibility and capacity of cells to utilize glucose and fatty acids. This cellular mitochondrial defect may be at least partially due to the decreased viscosity of cellular membranes that limits the activity of many rate-limiting metabolic processes in ER, mitochondria, and Golgi complexes^60,61^. Accordingly, the daily intake of glucose and fatty acids may not be effectively metabolized during feeding and fasting cycles due to disrupted mitochondrial function.

Alternatively, the rigid fluidity of the plasma membrane associated with 12-h clock dysfunction via *Xbp1* ablation may also negatively affect TG transportation to peripheral tissues via VLDL^36,37,56,57^. Indeed, previous studies have demonstrated that ablation of *Xbp1* in the liver resulted in a profound reduction of TG transportation via VLDL, in the absence of changes in ER protein secretion or the levels and content of plasma apolipoprotein B (apoB) proteins^27,28^. This defined phenotype may not be noticeably manifested in the relatively younger mice, as the ablation of *Xbp1* also profoundly reduces de novo hepatic lipid synthesis^27^. Also, the basal metabolic rate is generally higher in younger mice and decreases in an age-dependent manner^62,63^. Thus, the daily fat accumulation resulting from reduced cellular metabolism may not be efficiently transported to other peripheral tissues for storage or utilization, thus gradually accumulating in the liver. This mechanism could at least partially explain the spontaneous NAFLD phenotype that manifests chronically in an age-dependent manner, which we demonstrate upon disruption of the 12-h clock via *Xbp1* ablation under normal chow conditions.

While several different factors, such as temperature and cholesterol composition, also affect membrane fluidity^36,37,48,55^, our metabolomic assessment of liver metabolites led us to identify an unexpected role of the 12-h clock in the fatty acid monounsaturated and phospholipid remodeling pathways that are controlled by the rate-limiting metabolic enzymes including SCD1, LPCAT3, and LCAT. Accordingly, it has been shown that SCD1, LPCAT3, and LCAT activity correlates with membrane saturation^23,54,64–66^, wherein elevated ER stress was observed upon loss of either SCD1 or LPCAT3 activity^23,54,64^, and was associated with an increased level of LCAT in animal models of either obesity or overnutrition^65,66^. Thus, the impacts of SCD1, LPCAT3, and LCAT on membrane fluidity have been proposed as secondary effects linked to ER stress responses^23,54,64–67^. In contrast, we observed neither any noticeable apoptotic or inflammatory features, nor liver functional damage in the NAFLD phenotypic status resulting from hepatic *Xbp1* ablation. Consistent with our observation, previous studies have extensively shown that *Xbp1* ablation in the mouse liver causes minimal ER stress^27,28^, suggesting that the impact of XBP1-dependent 12-h clock on dynamic membrane fluidity may not depend upon classical ER stress responses. Instead, we show a direct 12-h temporal regulation of the key enzymes SCD1, LPCAT3, and LCAT in the fatty acid monounsaturated and phospholipid remodeling pathways. Thus, our study provides insights into 12-h clock regulation of membrane lipid composition and fluidity that are separable from ER stress responses in the setting of metabolic disease.

This link of 12-h clock and membrane fluidity in the maintenance of metabolic homeostasis may have implications for a type of human NAFLD that arises in the absence of obesity and detectable ER stress responses and under normal dietary conditions. For example, night-shift workers who ‘periodically’ change their night and day shifts and people who travel overseas not only alter their circadian behavior, but often change their feeding behavior and food metabolism^68,69^, both of which have been proposed to contribute to the regulation and entrainment of 12-h rhythms^1,4^. Accordingly, disruptions to both the 12-h clock and circadian clock mechanisms may lead to the development of fulminant NAFLD and type 2 diabetes mellitus.

While our findings suggest an important role of the 12-h clock in NAFLD development, this work does have some limitations. The XBP1-dependent 12-h clock is linked to membrane fluidity and lipid homeostasis, but how the 12-h oscillating genes disrupt plasma and mitochondrion membrane fluidity per se, and how these factors impact mitochondrial function and the selectivity of metabolism of glucose and fatty acids without impacting glutamine utilization is unclear. XBP1 may also have non-rhythmic mechanisms of action and some effects from aging that affect NAFLD development. While we believe there may be an unappreciated but important link between the 12-h clock and aging and/or aging-associated diseases, e.g., Alzheimer’s, we did not use any aging mice (at least over 18-24 months of age) or aging models. All mice in this study were between 15 weeks and 60 weeks of age under normal chow conditions. We did not observe any noticeable aging morphology under the above-mentioned conditions. Also, we did not use any ‘special’ diet in this study. This study was performed using only male animals, and future studies using female animals should be performed to investigate the potential effects of gender on the XBP1-regulated 12-h clock. XBP1s transcriptionally regulates the hepatic 12-h clock to coordinate liver glucose and lipid homeostasis, but more causal links between the 12-h clock and NAFLD susceptibility, including detailed characterizations of the 12-h clock and its harmonics with the 24-h clock in other conditions, e.g., prolonged constant darkness, feeding or behavior changes that disrupt the 12/24-h rhythmicity, and exploration of additional 12-h clock regulators in central and peripheral systems remain to be investigated.

In summary, our findings underscore an intimate and unappreciated relationship between the 12-h clock and the maintenance of metabolic homeostasis through the control of lipid homeostasis and the synchronization of systemic EE. Our data support the notion that disruption of systemic metabolism via genetic perturbations to the hepatic 12-h clock is at least partially driven by mechanisms associated with the PC-LPC cycle and unsaturated phospholipid pathways and the maintenance of membrane fluidity. We further substantiated the existence and importance of XBP1s as a key transcriptional regulator of the 12-h clock that directly regulates hepatic 12-h gene expression to coordinate metabolic and stress rhythms. Unlike the circadian clock that is imposed by the solar day, 12-h feeding behaviors likely originated during the evolution of ancient sea animals, which was likely entrained by 12-h tidal cues that are orchestrated mainly by the lunar cycles^2,4^. In this context, metabolism linked to these 12-h cycles may act as a tidal metabolic stressor that provided the original entrainment cues for the circatidal 12-h clock. Also, our comprehensive 12-h clock transcriptomic and cistromic analyses of mouse liver provide convincing evidence that XBP1 is a bona fide transcriptional regulator of the 12-h clock. Clinical evidence further indicates that perturbations in 12-h gene expression are associated with NAFLD progression and many other metabolic disorders in human patients. Our studies have uncovered an unexpected role of the hepatic 12-h clock and its metabolic output as a potential molecular target to more precisely understand, prevent, and treat metabolic disease.

## Methods

### Animals

*Xbp1*^*flx/flx*^ mice were kindly provided by Dr. Xi Chen at Baylor College of Medicine^27^. *Xbp1*^*flx/flx*^ mice were crossed with *AlbCre* transgenic mice (Jackson Laboratories) to generate *AlbCre; Xbp1*^*flx/flx*^, as well as *Xbp1*^*flx/flx*^ and *AlbCre* littermate controls. All offspring from this cross were maintained on a C57BL/6 background. Mice were maintained on a 12 h:12 h light:dark cycle and allowed free access to regular chow and water under strict temperature control. This study was conducted in male mice, not female mice. All animal studies were approved by the Institutional Animal Care and Use Committee (IACUC) at Baylor College of Medicine (BCM) and conducted in accordance with the BCM Core Animal Facility’s guidelines. All mice were bred in our animal facility at the BCM, and have complied with all relevant ethical regulations.

### Circadian time (CT) animal preparation and organ collection

Male *Xbp1*^*flx/flx*^ and *AlbCre Xbp1*^*flx/flx*^ mice between 12 and 16 weeks of age were entrained to a 12 h:12 h light:dark schedule for 14 days (the subjective day was beginning at 06:00 (ZT0) with subjective night beginning at 18:00 (ZT12), respectively), then released into constant darkness for 24 h. After the first 24 h continuous darkness, mice were euthanized at selected CT times in constant darkness, and tissues collected and snap-frozen in liquid nitrogen every two hours for 48 h. Food and water were supplied ad libitum at all stages prior to euthanization.

### Zeitgeber time (ZT) animal experiments

For experimental chronology measured in ZT, the subjective day was in a 12 h:12 h light:dark schedule, beginning at 07:00 (ZT0) with subjective night beginning at 19:00 (ZT12), respectively.

### RNA-Seq data

RNA samples were isolated from two biological replicates of mouse liver tissues from CT0 every 2 h for 48 h with the miRNeasy Mini Kit (Qiagen) per the manufacturer’s instructions. These RNA samples (200 ng of total RNA for each sample) were converted into strand-specific total mRNA-Seq libraries with the Universal Plus mRNA-Seq kit (NuGen) per the manufacturer’s protocol. The size selected libraries were analyzed for quality control with high Sensitivity DNA chip on a Bioanalyzer 2100 (Agilent), and the pooled libraries were sequenced with the NoveSeq 6000 (Illumina) using a 100-bp paired-end for an average of 40 million paired-end reads per sample at the University of Houston Sequencing Core.

### RNA-Seq analysis and oscillation detection

Fastq files containing raw RNA-Seq reads were aligned to the mouse genome (mm10/NCBI38) using HISAT2 (2.1.0). RNA-Seq quantification was performed with htseq-count (0.9.1, default parameters). Protein-coding genes were quantified by iGenome annotation (archive-2015-07-17-14-33-26). Quantification values were normalized with DESeq2 (2.11.40.2), followed by filtering out of zero expression genes in each replicate or time point. The mean values of normalized RNA-Seq data at each time point were used to determine the superimposed oscillations via the eigenvalue/pencil method^4,29^. In brief, the eigenvalue/pencil analysis of each gene resulted in three superimposed oscillations. We used the same period criterion established by Hughes et al.^1^ for circadian genes and 12-h genes for consistency and for better comparison and understanding of the prevalence of 12-h genes: circadian genes (the period between 20 and 28 h), 12-h genes (period between 10 and 14 h). Matlab_R2019A was used to determine the eigenvalue/pencil superimposed oscillations. The established rhythmic algorithms such as JTK_CYCLE and ARSER have a period pre-assignment bias, and thus are not capable of characterizing all superimposed oscillations^1,4^. Therefore, we chose to use the eigenvalue/pencil method to provide unbiased detection of both 12-h and 24-h oscillations in transcript abundance^4,29^. To ensure the robustness of the eigenvalue/pencil findings, we also used an independent RAIN method for validation^30^. RAIN as a non-parametric method for the detection of rhythms of prespecified periods and of arbitrary wave forms has been established for robustly identifying both circadian and ultradian rhythms, with one major limitation of its inability to identify multiple oscillations from each gene^4,29^. RAIN package in Bioconductor (3.4) (http://www.bioconductor.org/packages/release/bioc/html/rain.html) was used per default parameters^30,70^.

### ChIP-Seq data

Livers from the male *Xbp1*^*flx/flx*^ and *AlbCre*;*Xbp1*^*flx/flx*^ mice were harvested at the indicated CT time points, and two biological replicates were used for subsequent ChIP-Seq analysis. Mouse liver chromatin was prepared with the ChIP-IT Kit (Active Motif) per the manufacturer’s instruction with the following modifications. In brief, mouse liver samples were dissected into small pieces in PBS containing 1% formaldehyde and incubated for 15 min at room temperature, followed by the addition of 0.125 M glycine to stop the reaction. The formaldehyde-fixed samples were then washed in PBS, followed by homogenization in lysis buffer. The released nuclei were pelleted and resuspended in digestion buffer for 5 min at 37 °C. An appropriate amount of micrococcal nuclease (MNase) (NEB) was then added to enzymatically digest the chromatin. The optimal amount of incubation time was pre-determined to shear the chromatin into a 150-1000 bp banded pattern, and the reaction was stopped by 0.5 M EDTA, followed by chilling on ice for 10 min. Following chromatin quantification, an aliquot of 100 μg of mouse liver chromatin and 10 μg of anti-XBP1s antibody (Biolegend Poly6195) was used for the two CT0 and CT8 time points, with appropriate input genomic DNA saved for the comprehensive analysis. For the set of XBP1s ChIP-Seq from CT0 every 4 h for three complete 12-h cycles, an aliquot of 30 μg chromatin and 4 μg anti-XBP1s antibody was used. The co-immunoprecipitated complexes were then washed and eluted with elution buffer. The genomic DNA input and ChIP DNA fragments were reverse crosslinked at 65 °C overnight, and treated with RNaseA and proteinase K, and then purified by phenol-chloroform extraction and ethanol precipitation. The comprehensive CT0 and CT8 DNA libraries were prepared at the MD Anderson Cancer Center, which generated ~40 million 75 bp paired-end reads per sample. The CT0 to CT36 time point DNA libraries were prepared at the University of Houston Sequencing Core and generated ~30 million 75 bp single-end reads per sample.

### ChIP-Seq analysis

ChIP-Seq sequencing reads were mapped to the mouse genome (mm10/NCBI38) with BOWTIE2 (2.3.4.2). The mapping allowed a maximum of two mismatches and removed duplicated reads to ensure only “unique” reads were allowed for output to the bam file. Unique reads with mapping scores higher than 15 were kept for subsequent ChIP-Seq analysis. MACS2 version 2.1.1.20160309 was used to perform the peak calling at CT0 and CT8 against their respective input for each replicate individually, and only intersecting peaks between the two biological replicates at each time point was determined as high-confidence peaks. For the CT0 to CT36 4-h interval ChIP-Seq, duplicates at each time point were pooled, and heatmap and enrichment plotting were carried out against the ChIP-Seq of the *AlbCre;Xbp1*^*flx/flx*^ mice. In all the sequencing analysis, the differential sequencing reads were “down-sampled” to the lowest number of uniquely mapped reads for the normalization of sequencing depth. The ChIP-Seq peak annotations and motif analysis from the high-confidence peaks were determined using HOMER (v4.10.3) with default parameters.

### Gene ontology and disease perturbation analysis

Gene Ontology analysis was performed using DAVID (https://david.ncifcrf.gov). Biological process relationships and disease perturbations were performed. KEGG_PATHWAY analysis was used as the primary pathway category, and *Mus musculus* was set as background.

### Metabolic profiling using CLAMS

Comprehensive Lab Animal Monitoring System Calorimetry (CLAMS, Columbus Instruments) was used for real-time measurement of RER and food intake. Mice fed ad libitum were acclimated to the chambers for one week prior to data collection. Food intake was monitored for five consecutive days under a 12 h light:12 h dark cycle or constant darkness conditions as specified. CalR (version 1.2, https://calrapp.org) was used for the analysis of experiments using indirect calorimetry to measure physiological energy balance^58^. Data for VO_2_ consumed and VCO2 released were monitored, and RER was calculated as VCO_2_/VO_2_.

### Body composition analysis (via MRI)

The EchoMRI-100™ (Echo Medical System, Houston, TX), which is a nuclear magnetic resonance system, was used for measurement of whole-body fat mass, lean tissue mass, free fluid, and total body water without the need for anesthesia per the manufacturer’s instruction.

### Locomotor activity monitoring

Real-time measurement of spontaneous locomotor activity was performed with the Home Cage Activity System (Omnitech Electronics, Inc) in a home-cage environment. Mice fed ad libitum were acclimated to the home cage for at least one week prior to data collection. Spontaneous locomotor activity was measured under either 12 h light:12 h dark condition.

### Gene set enrichment analysis

GSEA with default phenotype permutation option was used for 12-h gene set enrichment against human hepatic steatosis and healthy steatosis microarray datasets obtained from GSE48452^31^. The array-based mRNA expression profiling of liver samples from NAFLD (*n* = 9) and NASH (*n* = 17) patients with healthy obese (*n* = 16) and controls (*n* = 12) was used for the GSEA analysis.

### Immunoblot

Immunoblot analyses were performed using the total liver extracts and SDS/PAGE. In brief, proteins separated by 4–20% gradient SDS/PAGE were transferred to nitrocellulose membranes, blocked in tris-buffered saline with Tween-20 supplemented with 5% (wt/vol) bovine serum albumin (BSA) and incubated with anti-XBP1s antibody (Biolegend Poly6195, 0.5 µg/mL) rotating at 4 °C overnight. Blots were incubated with an appropriate secondary antibody coupled to horseradish peroxidase (Digital anti-Rabbit-HRP, R1006, 1:1000, Kindle Biosciences), reacted with ECL reagents per the manufacturer’s instructions (Thermo), and detected by autoradiography or KwikQuant Imager (Kindle Biosciences).

### Mouse embryonic fibroblasts

MEFs were isolated from male *Xbp1*^*flx/flx*^ mice and immortalized by transfection with the *SV40* Large T antigen^4^. For *Ade-Cre* treatment, MEFs were infected with either adenovirus harboring an expression cassette for *CRE* recombinase or *CRE-GFP* and cultured in DMEM (4.5 g/L glucose) supplemented with 10% FBS at 37 °C with 5% CO_2_.

### Primary hepatocytes

Primary hepatocytes were isolated from male *Xbp1*^*flx/flx*^ and *AlbCre;Xbp1*^*flx/flx*^ littermate mice (*n* = 3), pooled and plated on 96 wells. Primary hepatocytes were cultured overnight in in Williams E media (Invitrogen) containing 10% FBS before membrane fluidity assays.

### Plasma profiling

Mouse plasma glucose levels were measured with a handheld glucometer, and mouse plasma insulin was measured with a mouse insulin kit (Millipore). Mouse plasma TG, TC, and liver function (i.e., AST/ALT) parameters were measured by the mouse Metabolism Core Services at Baylor College of Medicine.

### Glucose and ITTs

To determine glucose tolerance, mice were fasted for 16 h, and glucose was administered (1.5 g/kg body weight) by intraperitoneal injection. To determine insulin tolerance, mice were fasted for 6 h prior to intraperitoneal injection of insulin (0.75 units/kg body weight). Blood glucose levels were measured using a handheld glucometer, and mouse plasma insulin was measured with a mouse insulin kit (Millipore).

### Metabolomics profiling

Metabolite concentrations were obtained using the AbsoluteIDQ kit p180 (Biocrates Life Science AG, Austria) according to manufacturer’s instructions on an QTRAP 6500 LC/MS/MS System (AB SCIEX, USA) equipped with an electrospray ionization source, an Agilent G1367B autosampler and the Analyst 1.51 software (AB SCIEX, USA). In brief, 2 mg of tissue lysate (20 μl) of mouse whole liver samples were pipetted onto the spots of the kit plate. Spots were dried at room temperature in a nitrogen evaporator drying unit for 30 min. Twnty microliters of 5% PITC reagent was pipetted onto the spots and incubated for 20 min at RT. The plate was dried under the nitrogen evaporator for 60 min, followed by the addition of 300 μl of 5 mM ammonium acetate in methanol to each well and incubated on a shaker (450 rpm) for 30 min. The plate was centrifuged at 100 × *g* for 2 min receiving about 250 μl sample in flow injection analysis (FIA) plate. Five hundred microliters of MS running solvent (Biocrates solvent diluted in methanol) was added to the FIA plate. Acylcarnitines, PC, LPC, sphingomyelins were measured using the FIA plate and detection of fragments in multiple reaction monitoring (MRM) mode of operation per manufacturer’s instructions. Twenty microliters of the sample was injected directly into the MS at a flow rate of 30 μl/min with MS running solvent. Concentrations were calculated and evaluated in the Analyst/MetIQ software by comparing measured analytes in a defined extracted ion count (XIC) section to those of specific labeled internal standards or non-labeled, non-physiological standards (semi-quantitative) provided by the kit plate. Column used: Agilent Zorbax eclipse XDB C18, 3.0 × 100, 3.5 µM. Buffers used for the analysis: 0.2% formic acid in water (A),0.2% formic acid in acetonitrile (B). The gradient used: Begins with 100% A up to 0.5 min, at 5.5 min 95% B, at 7 min 100% B min an equilibrated to initial gradient with a total runtime of 9.5 min.

### Metabolomics analysis

Analytes concentrations of Acylcarnitines, PC, LPC, sphingomyelins from the above FIA-MS/MS method were calculated and evaluated in the Analyst/MetIQ software by comparing measured analytes in a defined XIC section to those of specific labeled internal standards or non-labeled, non-physiological standards (semi-quantitative) provided by Biocrates. The raw concentration data were processed with R version 3.5.1 in a data file containing 16 liver samples (*n* = 4/group/age) by 146 analytes matrix format. Before data analysis, quantile normalization was performed to allow general-purpose adjustment for differences among samples; Log data transformation and mean-centered scaling and divided by the standard deviation of each variable were performed to make features more comparable. For multi-group univariate analysis, one-way analysis of variance (ANOVA) was performed to tell the important features with *p* value threshold 0.05, followed by post hoc analyses Fisher’s least significant difference method (Fisher’s LSD). Correlation analysis (spearman) was used to visualize the overall correlations between different features. Principal component analysis (PCA) as an unsupervised method was used with the prcomp package. The calculation is based on singular value decomposition. A discriminant analysis (PLS-DA) model-based permutation test was also performed to assess the significance of class discrimination with the plsr function provided by R pls package. Hierarchical cluster analysis was performed with the hclust function in package stat, shown as heatmap (distance measure using euclidean, and clustering algorithm using ward.D).

### Membrane fluidity assay

Membrane fluidity measurement was performed with the membrane fluidity kit per the manufacturer’s instruction (Abcam). In brief, the cultured cells (MEFs and primary hepatocytes) as indicated were perfused and then incubated in the labeling solution containing 10 μM PDA and a final concentration of 0.08% Pluronic F-127 with perfusion buffer at room temperature in the dark for 30 min. After washing in PBS, the cells were incubated in culture medium without pheno red for subsequent fluorescence reading. The cells were analyzed for fluorescence by excitation at 350 nm and emission at 405 nm and 460 nm, respectively.

### Mito stress and fuel flex metabolic assays

Mito stress and fuel flex tests were performed with Seahorse kits and the Seahorse XFe96 analyzer according to the manufacturer’s protocol (Agilent). In brief, cells were seeded in a Seahorse XF96 cell culture plate and incubated at 37 °C in a humidified 5% CO_2_ atmosphere overnight. Seahorse XF cartridges were hydrated with Seahorse XF calibrant and placed in a 0% CO_2_ incubator overnight. Prior to the assay, the culture medium was replaced with assay medium per the manufacturer’s instructions. Compound injections for different stress tests were prepared according to the manufacturer’s instructions (Seahorse XF Cell Mito Stress Test Kit and Seahorse XF Mito Fuel Flex Test Kit) and added to the respective ports on the cartridge. Three to five measurements were made for basal metabolism and each compound injection. Seahorse XF Report Generator (Agilent) was used to automatically calculates the key parameters of the Seahorse XF Cell Mito Stress and Mito Fuel Flex results per the manufacturer’s instruction.

### Immunohistochemistry

Fresh Liver tissues were fixed in 4% paraformaldehyde for 48 h and embedded in paraffin. Hematoxylin and eosin (H&E) staining was used to evaluate gross morphology, and Sirius Red was used to examine fibrosis. Antibodies specific to F4/80 (murine macrophage marker, Thermo Fisher Scientific, Cat#MF48000, 1:100) and Casp2 (apoptotic marker, Abcam Cat#ab2251, 1:200) were used to visualize macrophage infiltration and apoptotic features, respectively. Frozen-block preparation was performed by embedding liver tissues in OCT compound. Oil Red O staining was used to visualize neutral lipid accumulation.

### Statistics and reproducibility

Statistical analysis was conducted using GraphPad Prism 8 software. All data were tested for normal distribution of variables. All normally distributed data were displayed as the means ± standard error of the mean (SEM), unless otherwise noted. Measurements between two groups were performed with an unpaired Student’s *t*-test (two-tailed *p* value) unless otherwise noted. Non-significant *p* values are shown as ns. Each in vivo and in vitro experiment was performed with the number of replicates specified in the figure legends. All data obtained are shown; all data are shown in the quantification.

### Reporting summary

Further information on research design is available in the Nature Research Reporting Summary linked to this article.

## Supplementary information

Supplementary Information

Description of Additional Supplementary Files

Supplementary Data 1

Supplementary Data 2

Supplementary Data 3

Supplementary Data 4

Supplementary Data 5

Supplementary Data 6

Reporting Summary

## Data Availability

The genomic datasets generated in this study can be accessed at the GEO public repository using the accession number GSE150890. The array-based mRNA expression profiling of liver samples from NAFLD and NASH patients with healthy obese and controls were obtained from GEO GSE48452. The data that support the findings of this study are available in the source data files. All other relevant data are available upon reasonable request. Source data are provided with this paper.

## Source data

Source Data
