# Supplementary material for: XBP1 links the 12-hour clock to NAFLD and regulation of membrane fluidity and lipid homeostasis
Source: Nat Commun. 2020 Dec 4;11:6215. doi: 10.1038/s41467-020-20028-z (PMC7718229; doi:10.1038/s41467-020-20028-z)
Supplement: Supplementary file 1 — Supplementary Information [file 41467_2020_20028_MOESM1_ESM.pdf]

Supplementary Information

XBP1 links the 12-hour clock to NAFLD and regulation of membrane fluidity and lipid homeostasis

Meng et al.

Supplementary Figures 1 – 12

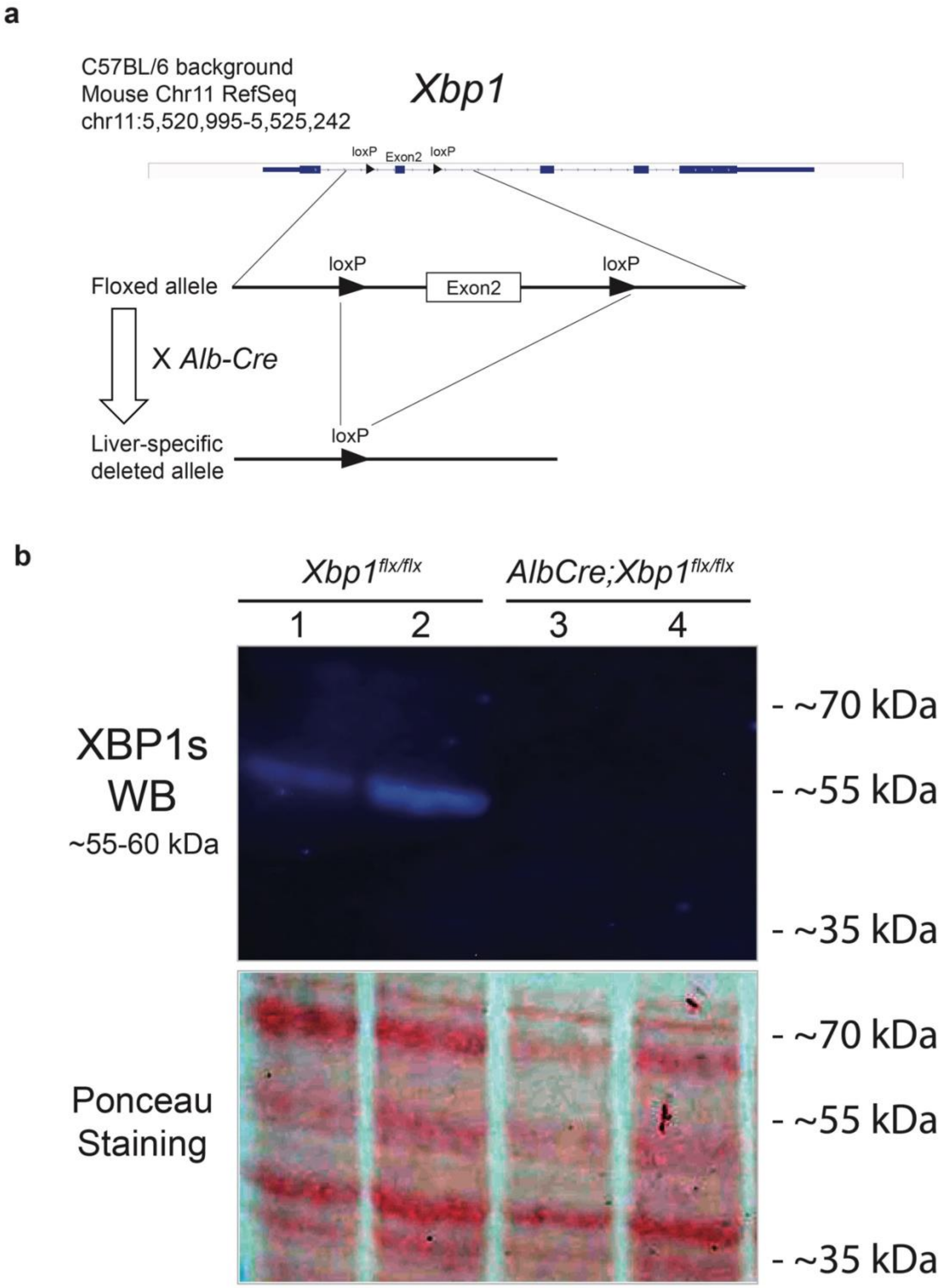
a
C57BL/6 background
Mouse Chr11 RefSeq
chr11:5,520,995-5,525,242
Xbp1
loxP
Exon2
loxP
Floxed allele
loxP
Exon2
loxP
X Alb-Cre
Liver-specific
deleted allele
loxP
b
Xbp1flx/flx
AlbCre;Xbp1flx/flx
1
2
3
4
XBP1s
WB
~55-60 kDa
- ~70 kDa
- ~55 kDa
- ~35 kDa
Ponceau
Staining
- ~70 kDa
- ~55 kDa
- ~35 kDa

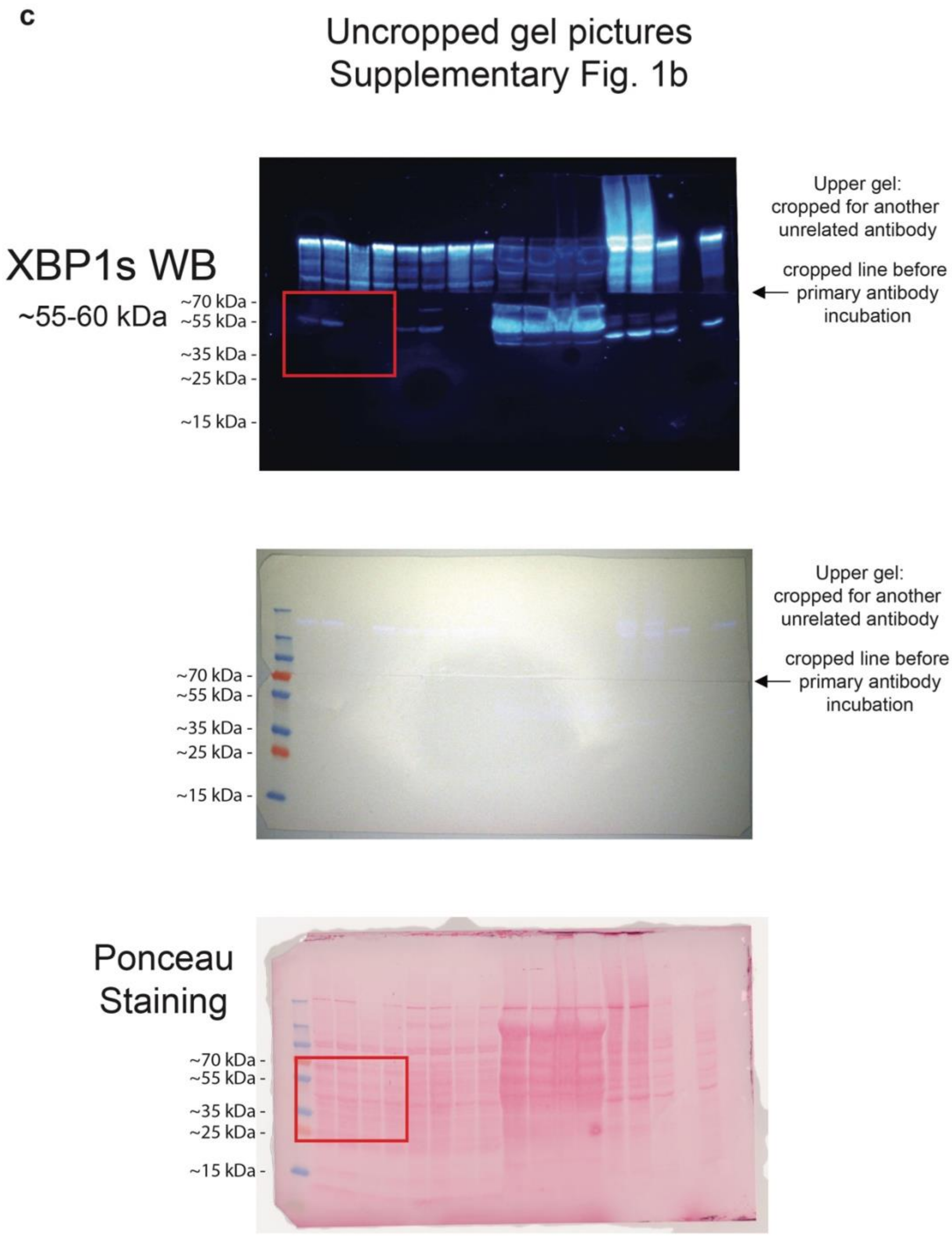

Supplementary Figure 1. Liver-specific deletion of *Xbp1* in mice. a. The strategy to delete *Xbp1* in a liver-specific manner. The generation of mice containing *Xbp1* exon2 flox allele (*Xbp1* $^{flx/flx}$) and liver-specific deletion of exon 2 (*AlbCre;Xbp1* $^{flx/flx}$) are shown. **b.** Immunoblot analysis of total hepatic XBP1s in the *Xbp1* $^{flx/flx}$ and *AlbCre;Xbp1* $^{flx/flx}$ mice. **c.** Uncropped gel and western blot pictures of Supplementary Figure 1b.

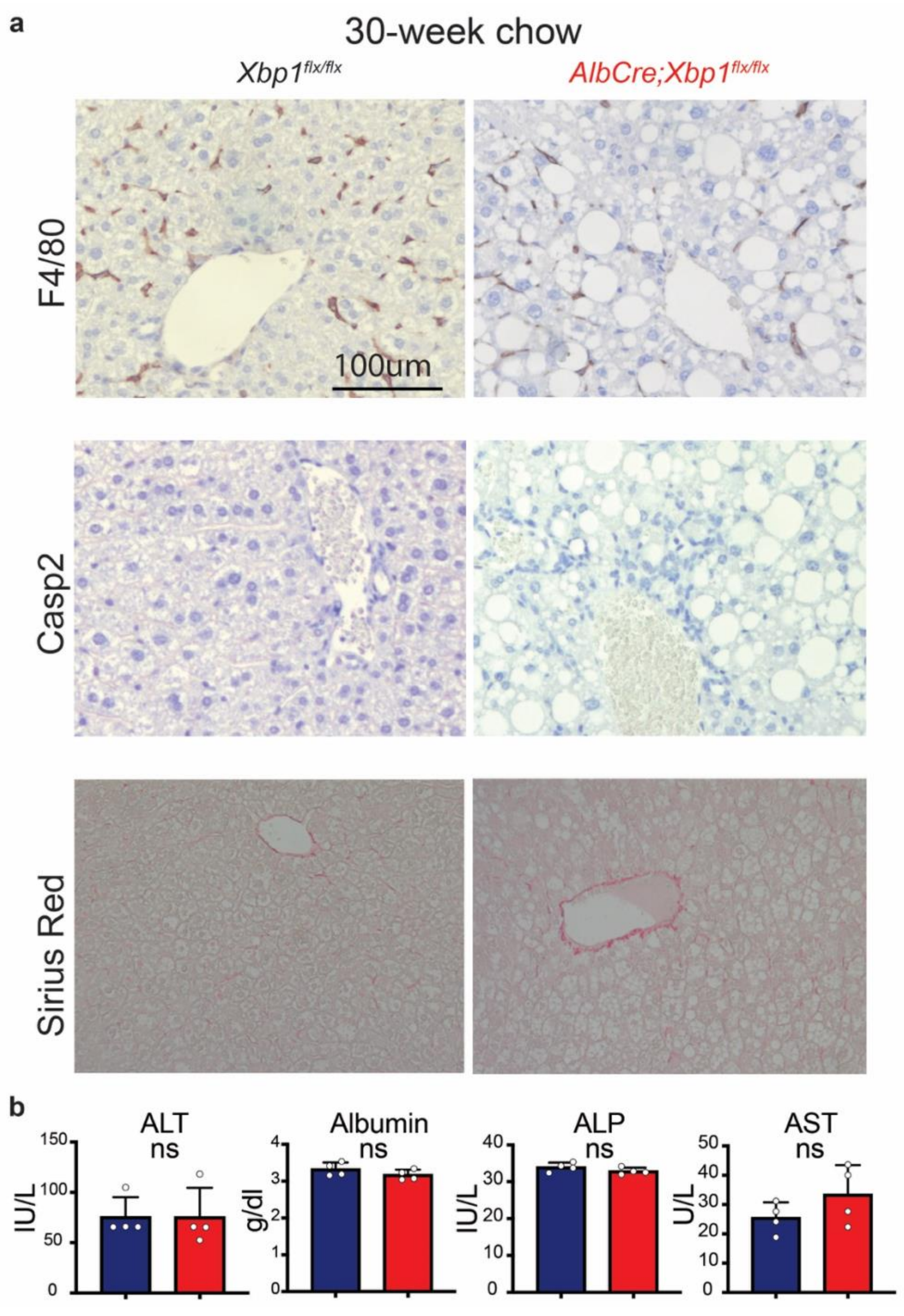

Supplementary Figure 2. Liver-specific deletion of *Xbp1* was not accompanied by any apoptotic or inflammatory features, nor by liver functional damage. Histological and liver functional mouse phenotyping features of the *Xbp1$^{flx/flx}$* and *AlbCre;Xbp1$^{flx/flx}$* mice fed regular chow *ad libitum* at 30 weeks of age are shown. **a.** F4/80, Casp2, and Sirius Red staining of liver sections from the indicated mouse strains did not show any noticeable liver morphological defects. **b.** Blood AST, ALT, ALP, and albumin of the indicated mouse strains (n = 4). Unpaired Student's t-test was performed with p value indicated. Data are graphed as the mean ± SEM.

a

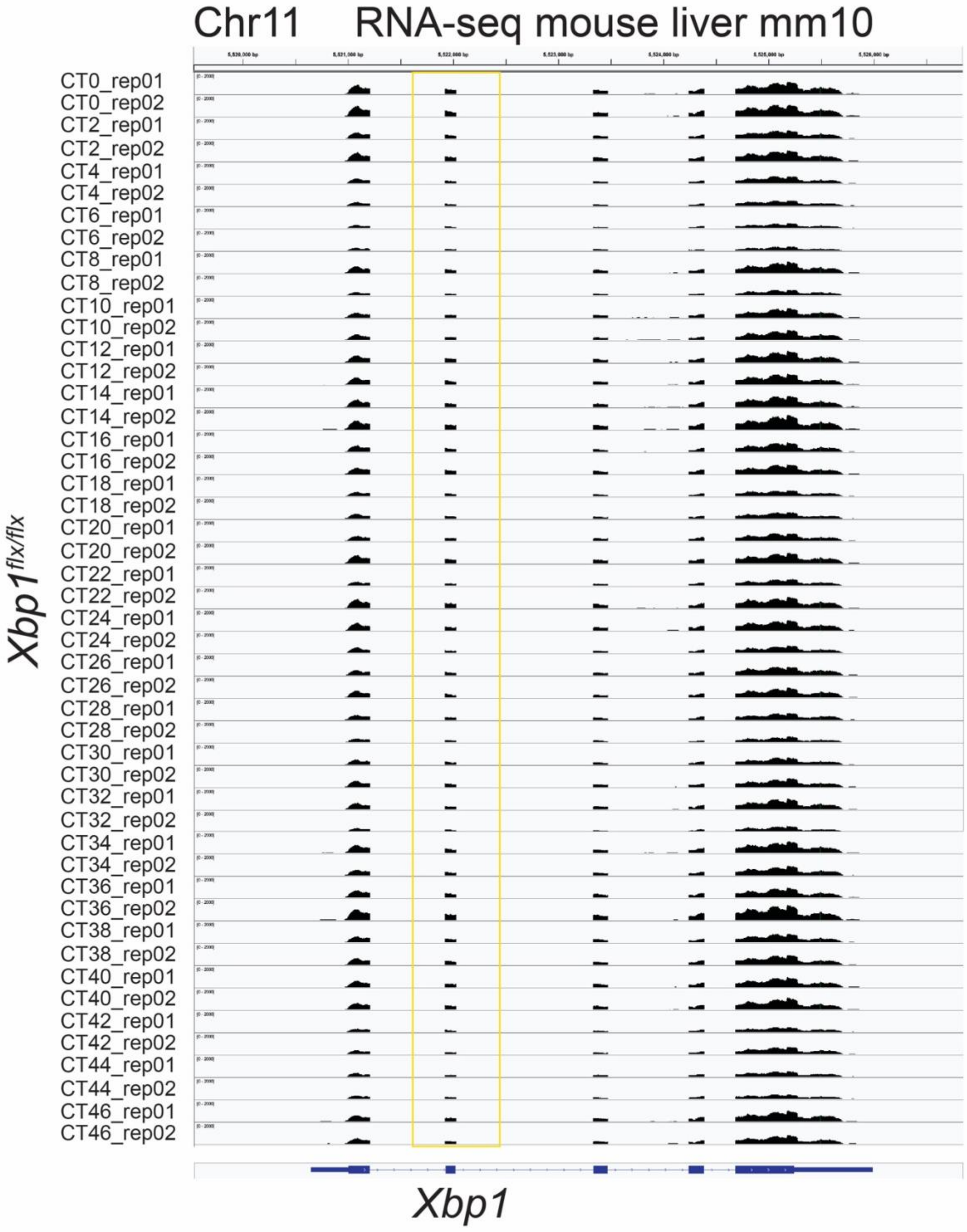
Chr11 RNA-seq mouse liver mm10
CT0_rep01
CT0_rep02
CT2_rep01
CT2_rep02
CT4_rep01
CT4_rep02
CT6_rep01
CT6_rep02
CT8_rep01
CT8_rep02
CT10_rep01
CT10_rep02
CT12_rep01
CT12_rep02
CT14_rep01
CT14_rep02
CT16_rep01
CT16_rep02
CT18_rep01
CT18_rep02
CT20_rep01
CT20_rep02
CT22_rep01
CT22_rep02
CT24_rep01
CT24_rep02
CT26_rep01
CT26_rep02
CT28_rep01
CT28_rep02
CT30_rep01
CT30_rep02
CT32_rep01
CT32_rep02
CT34_rep01
CT34_rep02
CT36_rep01
CT36_rep02
CT38_rep01
CT38_rep02
CT40_rep01
CT40_rep02
CT42_rep01
CT42_rep02
CT44_rep01
CT44_rep02
CT46_rep01
CT46_rep02
Xbp1 flx/flx
Xbp1

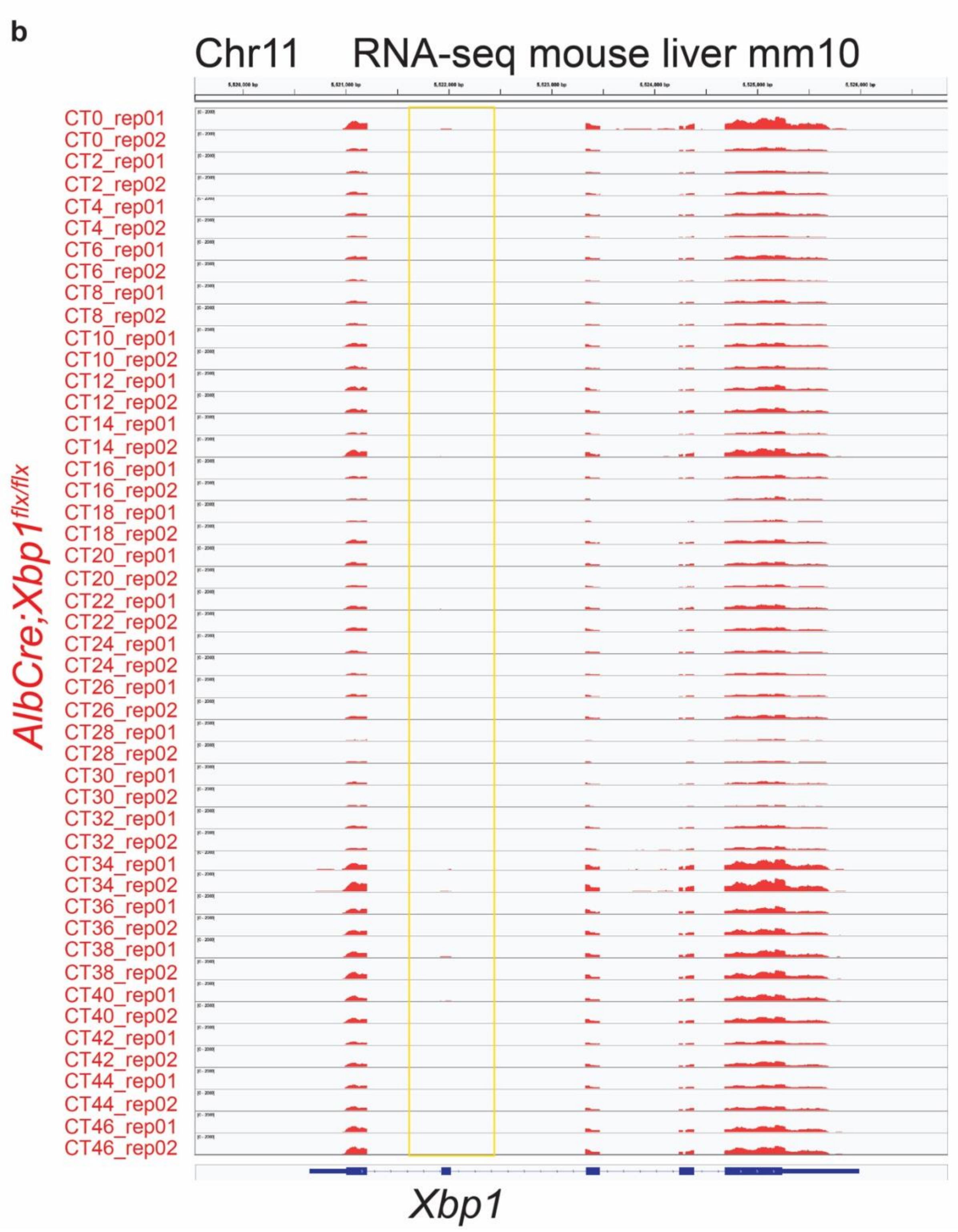

Supplementary Figure 3. Absence of *Xbp1* exon2 expression in RNA-Seq data of *AlbCre;Xbp1*$^{flx/flx}$ mice. Mouse hepatic mRNA views of all RNA-Seq data at the *Xbp1* locus of *Xbp1*$^{flx/flx}$ (**a**) and *AlbCre;Xbp1*$^{flx/flx}$ (**b**) mice are shown. Yellow square indicates the exon 2 of the *Xbp1* gene that is floxed with loxP sites.

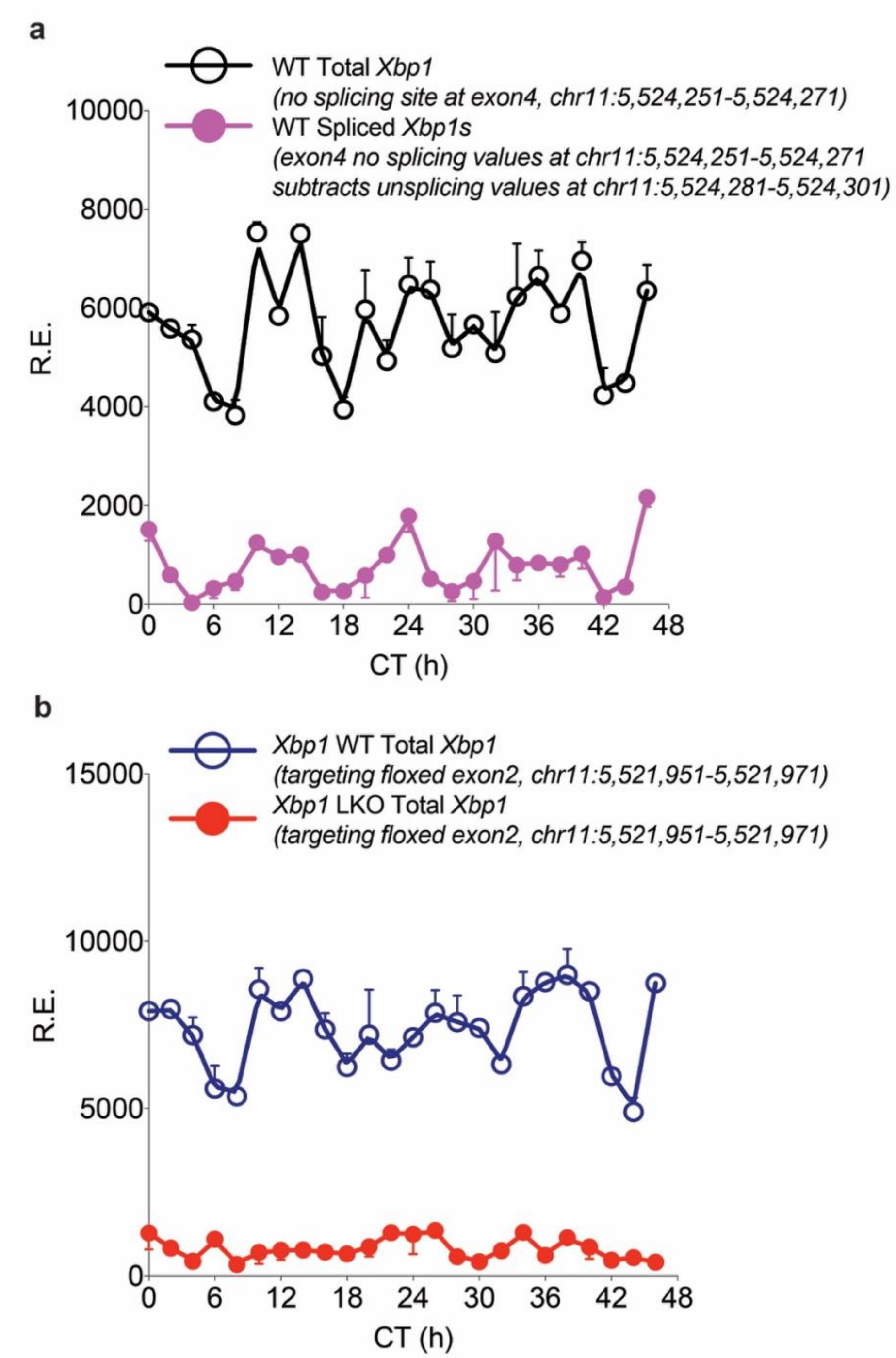

Supplementary Figure 4. Liver-specific ablation of *Xbp1* impairs hepatic 12-hour rhythmicity of total *Xbp1* and spliced *Xbp1s* gene expression. a. The 12-hour gene expression patterns of total hepatic *Xbp1* mRNA (black) and spliced *Xbp1s* mRNA (pink) that are detected by *in silicon* targeting exon 4 in $Xbp1^{flx/flx}$ mice (n = 2 per timepoint). **b.** Total hepatic *Xbp1* mRNA at exon2 that is floxed in the $Xbp1^{flx/flx}$ and $AlbCre;Xbp1^{flx/flx}$ mice (n = 2 per timepoint). The y axis represents the average signal in a 1-bp bin (normalized uniquely mapped reads) from the RNA-Seq data in Figure 2 and Supplementary Table 1. Data are graphed as the mean ± SEM.

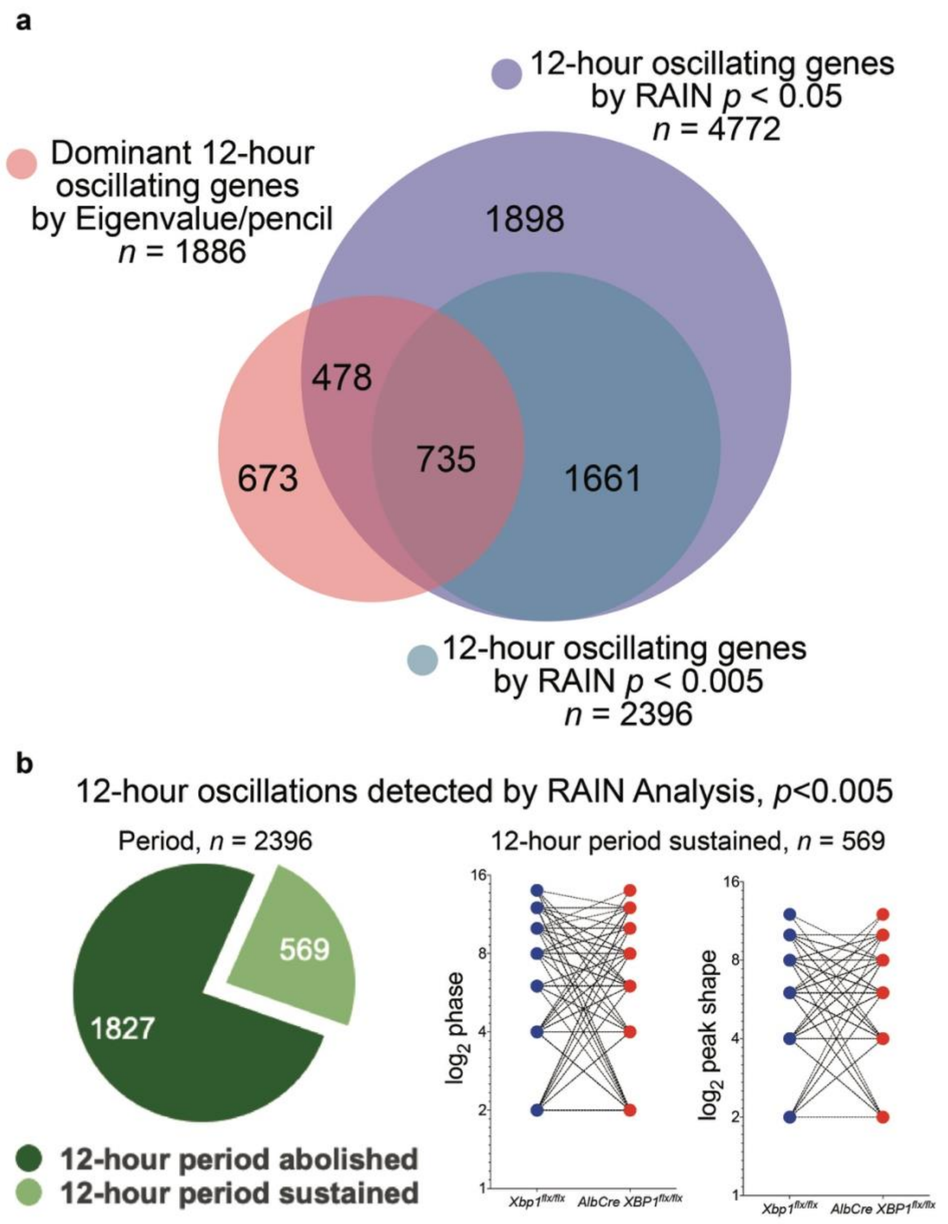

Supplementary Figure 5. The 12-hour oscillating genes detected from an additional statistical analysis of the primary RNA-Seq data by an independent rhythmicity analysis incorporating non-parametric method (RAIN). a. Venn diagram comparison of the 12-hour transcriptome detected by the eigenvalue/pencil method and RAIN method (with different FDR cut-offs of 0.005 and 0.05) in the *Xbp1*$^{flx/flx}$ mice. **b.** Left: the proportion of the 12-hour gene oscillations uncovered by RAIN method that had abolished (n = 1,827) or sustained (n = 569) 12-hour periods in the *AlbCre;Xbp1*$^{flx/flx}$ mice. Right: the matched-pairs of individual plots of phase and peak shape that were calculated by RAIN. The RAIN method tests whether the time course datasets consist of alternating rising and falling slopes, repeated with a distinct period. The partitions of the rising part with respect to the whole period are given by peak.border = c(min, max). The value peak.shape specifies this partition in the best matching model. The phase is defined as the time point with the peak.

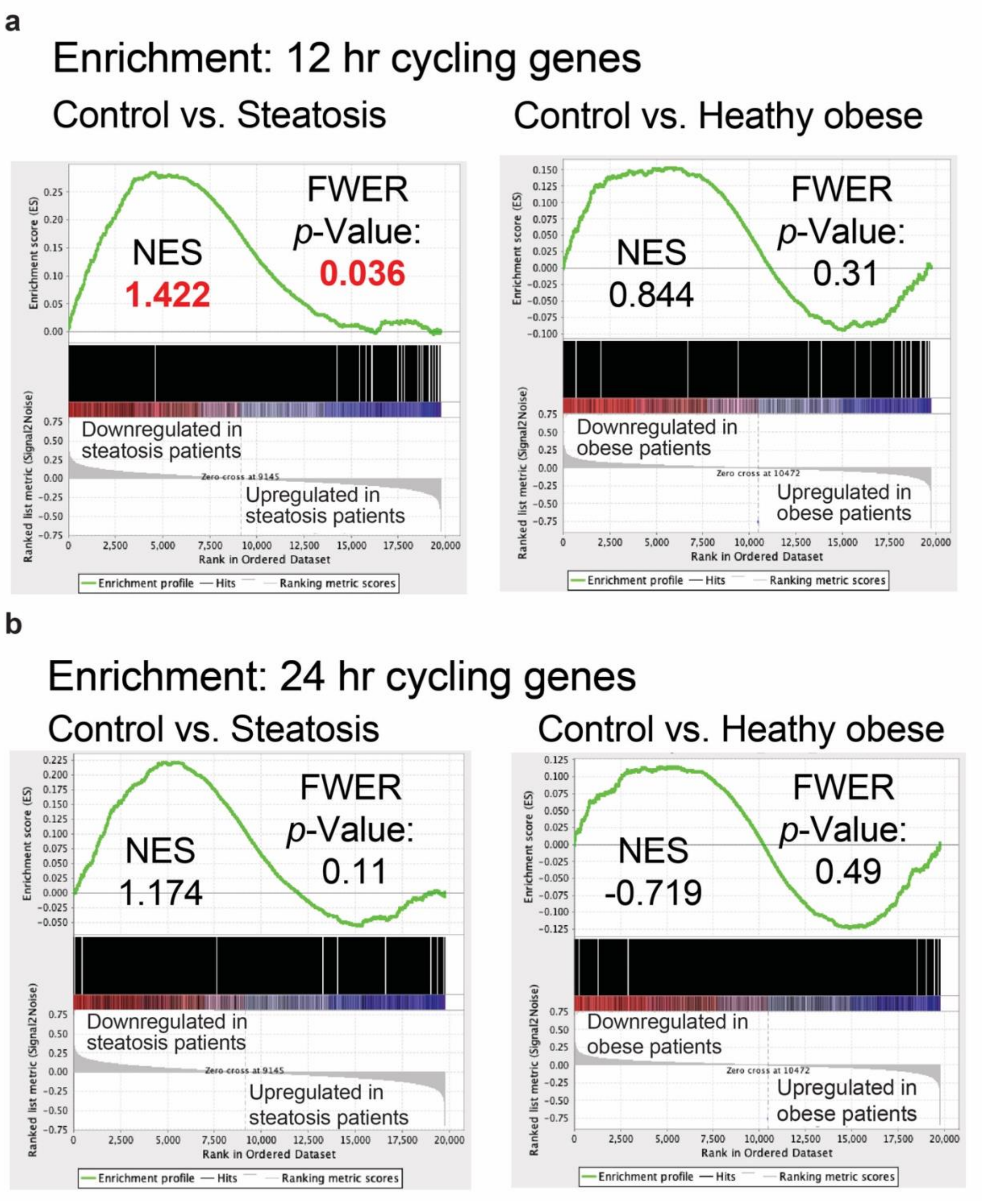

Supplementary Figure 6. Gene set enrichment analysis (GSEA) of the 12-hour and 24-hour cycling genes. Detailed gene set enrichment analysis (GSEA) of the 12-hour (**a**) and 24-hour (**b**) cycling genes enriched in human hepatic steatosis (left) and healthy obese (right) gene expression datasets (GSE48452). Normalized enrichment scores (NES), FWER *p* values, and rank in ordered metrics are shown.

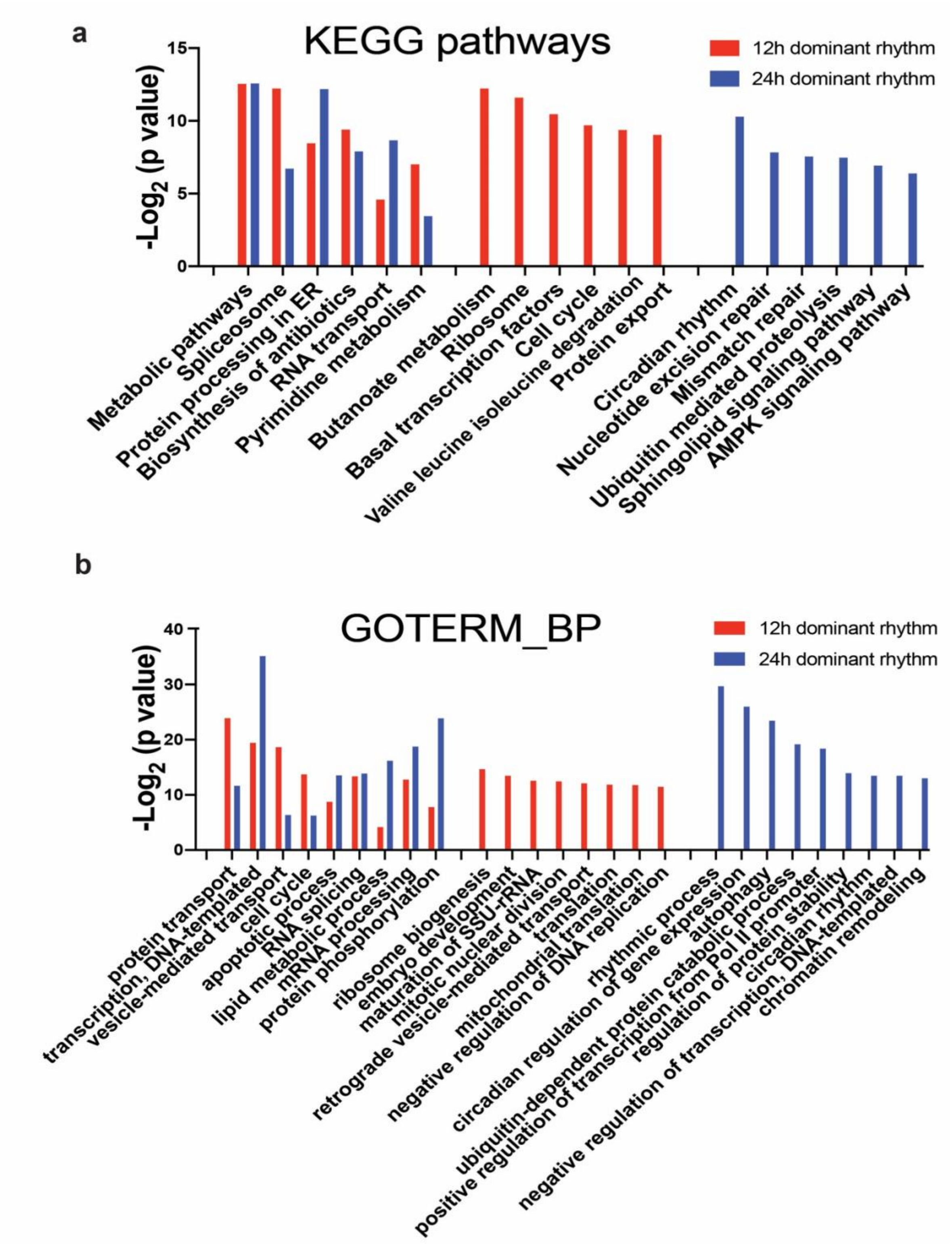

Supplementary Figure 7. Pathway analysis of 12- and 24-hour cycling genes. a. Top-enriched KEGG pathways identified in 12- and 24-hour cycling genes, respectively. **b.** Top-enriched GOTERM_BP terms identified in 12-hour and 24-hour cycling genes.

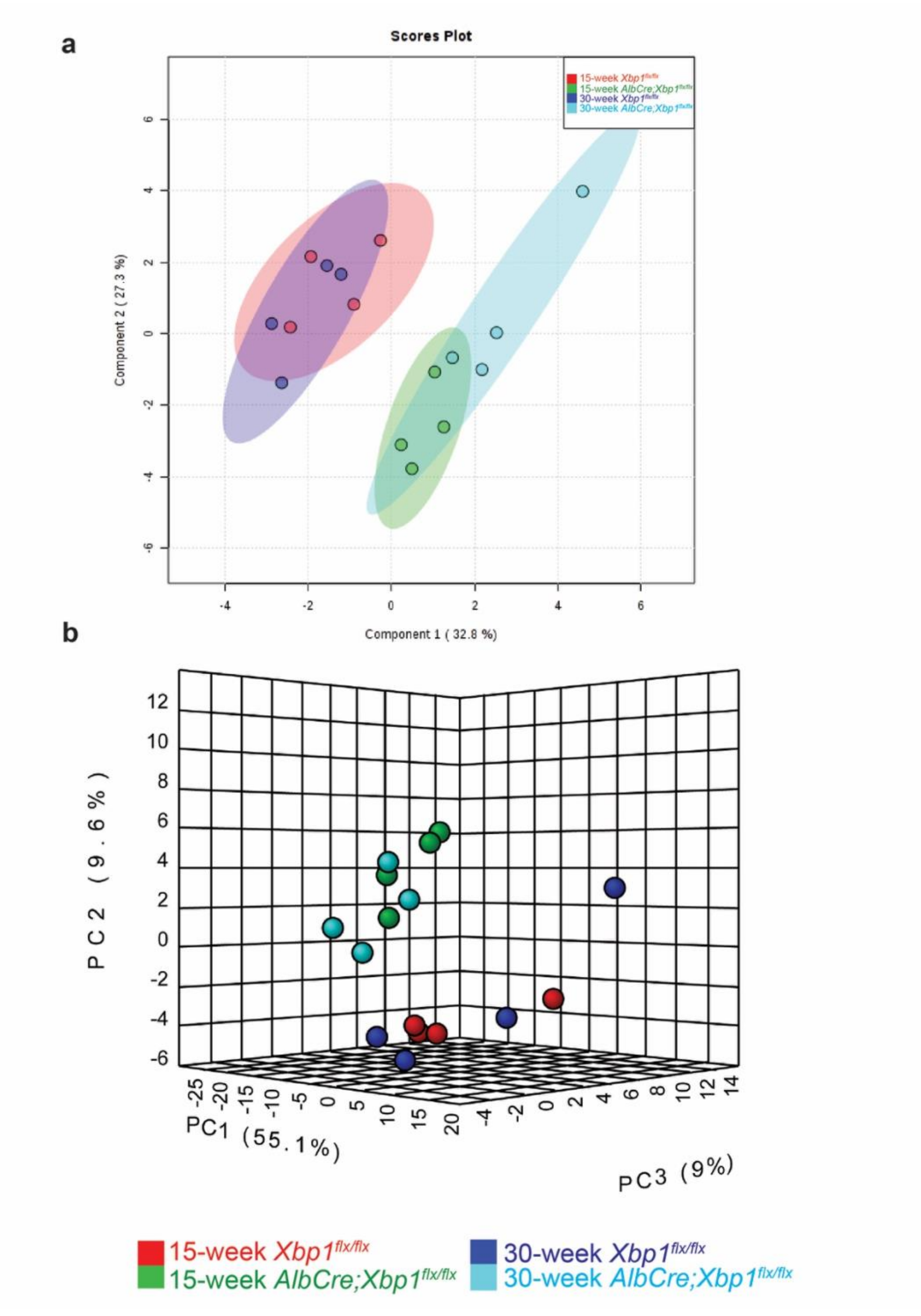

Supplementary Figure 8. Liver metabolomic profiling groups. a-b. Sparse Partial Least Squares - Discriminant Analysis (sPLS-DA) **(a)** and 3-D Principal Component Analysis (PCA) **(b)** shows the individual metabolomics data from the *Xbp1$^{flx/flx}$* and *AlbCre;Xbp1$^{flx/flx}$* mice fed regular chow *ad libitum* at 15- and 30-weeks of age (*n* = 4/group/age).

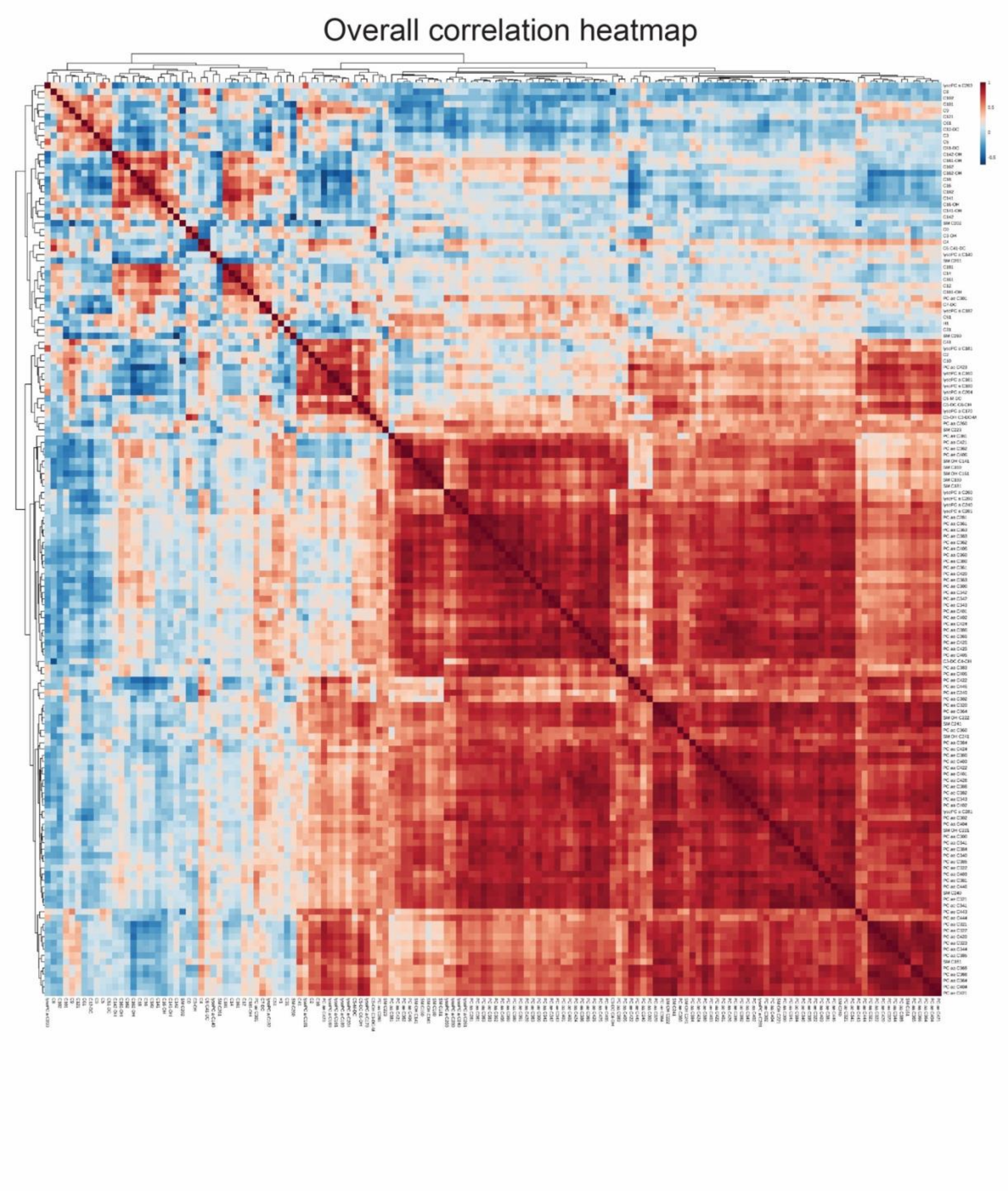

Supplementary Figure 9. Overall correlation heatmap of the liver metabolomic profiling. Correlation analysis was used to visualize the overall correlations between different features of the metabolomics data from the *Xbp1*$^{flx/flx}$ and *AlbCre;Xbp1*$^{flx/flx}$ mice fed regular chow *ad libitum* at 15- and 30-weeks of age (*n* = 4/group/age).

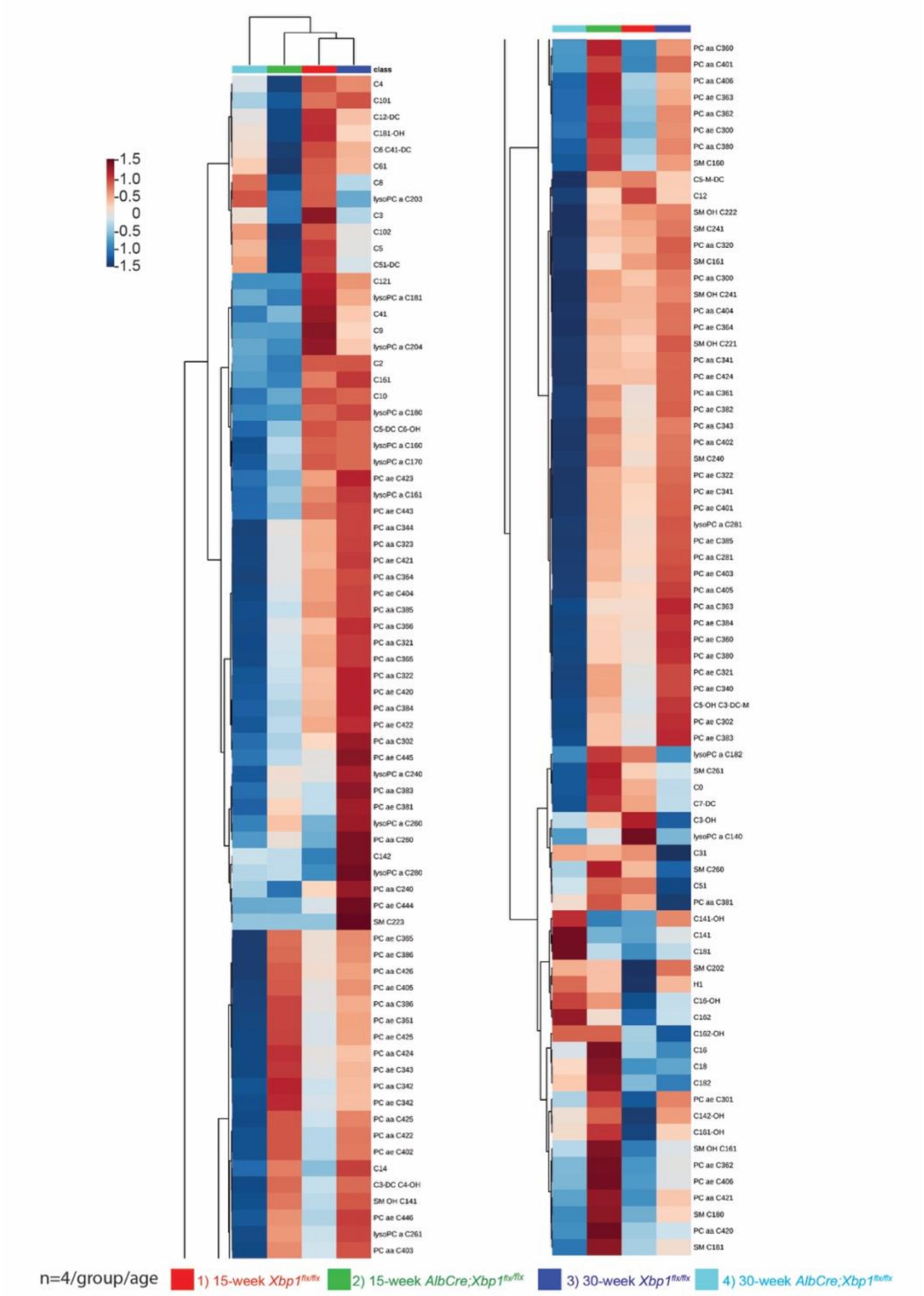

Supplementary Figure 10. Clustering heatmap of the liver metabolomic profiling. Hierarchical clustering heatmap analysis was used to visualize the similarity measurements and clustering results of the metabolomics data from the *Xbp1*$^{flx/flx}$ and *AlbCre;Xbp1*$^{flx/flx}$ mice (*n* = 4/group/age) fed regular chow *ad libitum* at 15- and 30-weeks of age (distance measure using euclidean, and clustering algorithm using ward.D).

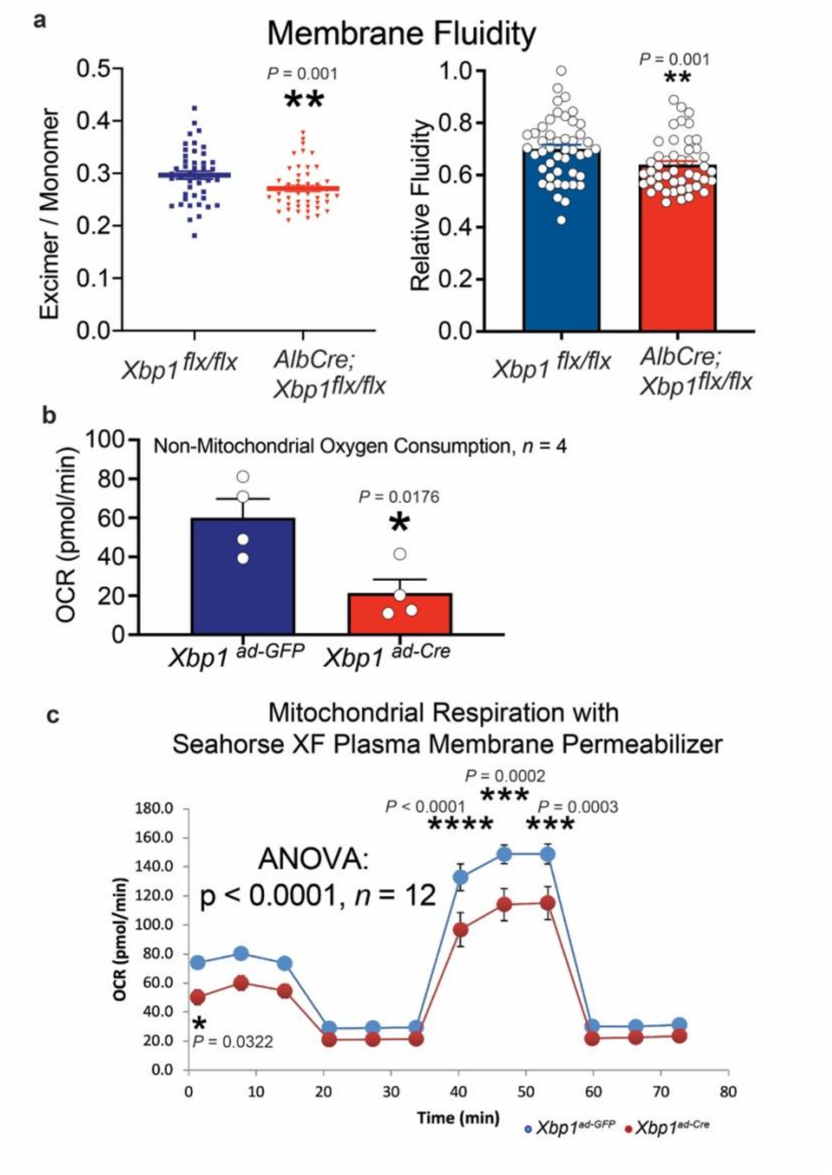

Supplementary Figure 11. *Xbp1* ablation significantly reduces cellular membrane fluidity, mitochondrial respiration, and non-mitochondrial oxygen consumption. a. Membrane fluidity of the indicated primary hepatocytes measured using pyrenedecanoic acid (PDA) (*n* = 46 independent 96-wells of primary hepatocytes per group). Primary hepatocytes were isolated from male $Xbp1^{flx/flx}$ and $AlbCre;Xbp1^{flx/flx}$ littermate mice (*n* = 3), pooled and plated on 96-wells. Experiments were repeated twice in biologically independent animals. The absolute excimer: monomer (left panel) and relative ratio (right panel) of emission at 460 nm to emission at 405 nm are shown. **b.** Non-mitochondrial oxygen consumption measured by Mito Stress tests in the indicated mouse embryonic fibroblasts (MEFs) strains is shown (*n* = 4 independent *Ad-Cre Xbp1* ablation). **c.** Cell Mito Stress test for mitochondrial respiration profile with seahorse plasma membrane permeabilizer is shown. Unpaired Student's t-test and one-way ANOVA analysis were performed with *p* value indicated. Data are graphed as the mean ± SEM. *p < 0.05, **p < 0.005, ***p < 0.001, ****p < 0.0001.

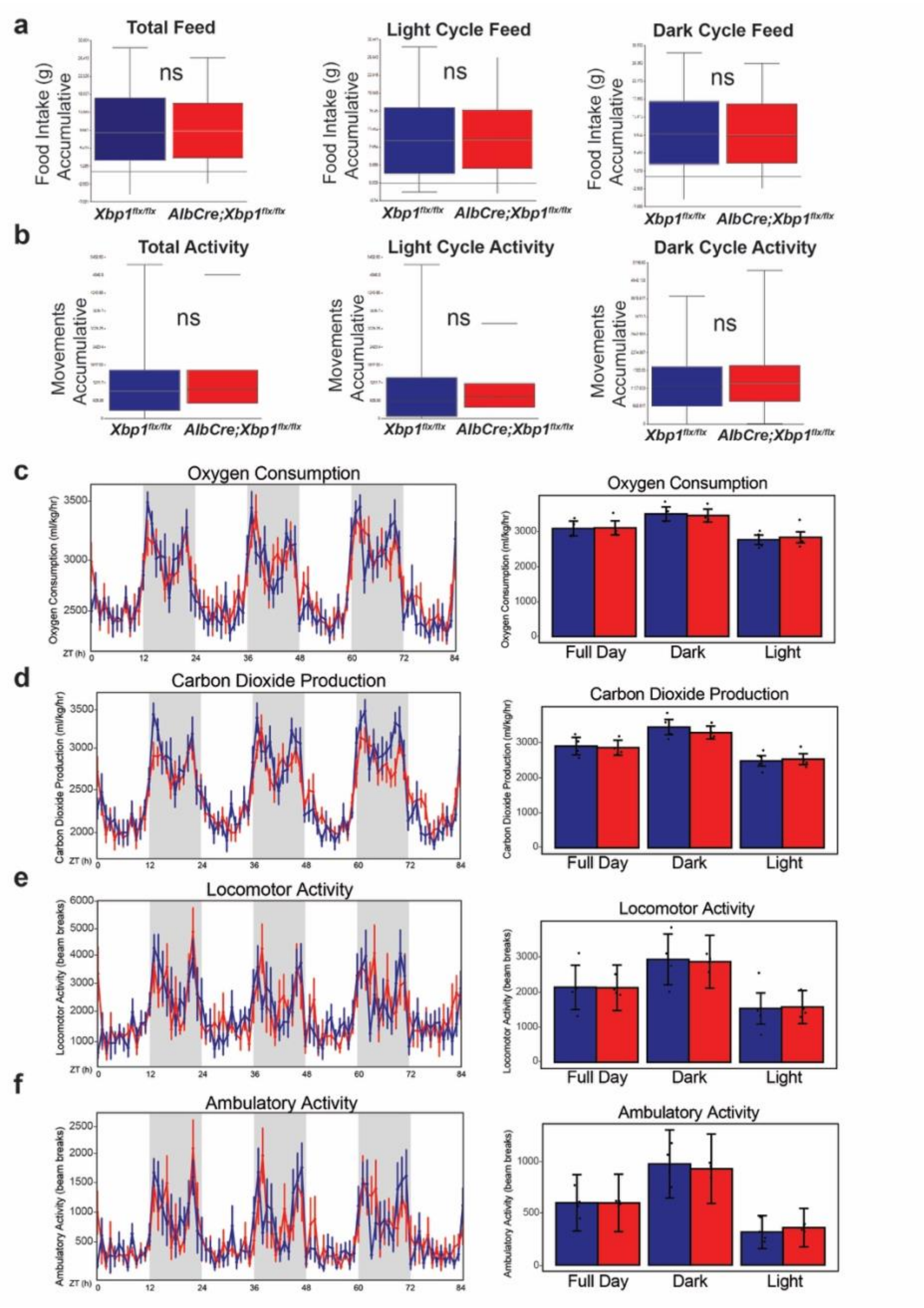

Supplementary Figure 12. Liver-specific ablation of *Xbp1* did not alter feeding behaviors, oxygen consumption, carbon dioxide production, and activity. a-b. Real-time measurement of food intake (**a**) and Real-time home cage activity (**b**) in the *Xbp1*$^{flx/flx}$ and *AlbCre;Xbp1*$^{flx/flx}$ mice fed regular chow *ad libitum* (*n* = 4). The bar plots show the normalized values (mean ± SEM). The boxes range from the 25% and the 75% percentiles; the 5% and 95% percentiles are indicated as error bars. Medians are indicated by horizontal lines within each box. **c-f.** The real-time raw values and statistics of mouse indirect calorimetry experiments for 12-hour rhythmicity of real-time oxygen consumption (**c**), carbon dioxide production (**d**), and locomotor (**e**) and ambulatory (**f**) activities (*n* = 4). Data are graphed as the mean ± SEM.
