# Supplementary material for: XBP1 links the 12-hour clock to NAFLD and regulation of membrane fluidity and lipid homeostasis
Source: Nat Commun. 2020 Dec 4;11:6215. doi: 10.1038/s41467-020-20028-z (PMC7718229; doi:10.1038/s41467-020-20028-z)
Supplement: Supplementary file 2 — Description of Additional Supplementary Files [file 41467_2020_20028_MOESM2_ESM.docx]

**Supplementary Data 1-6 Legends**

XBP1 links the 12-hour clock to NAFLD and regulation of membrane fluidity and lipid homeostasis

Meng et al.

**Supplementary Data 1.** **Next-Generation Sequencing (NGS) statistics for RNA-Seq (related to Figure 2) and ChIP-Seq (related to Figure 3 & 4).** The number of total and uniquely aligned reads for RNA-Seq & ChIP-Seq data are shown. (XLSX file)

**Supplementary Data 2.** **Normalized RNA-Seq quantification from the *Xbp1^flx/flx^* and *AlbCre;Xbp1^flx/flx^* mouse livers (related to Figure 2).** Gene Name, individual and mean quantification of all RNA-Seq samples at each circadian time (CT) point in the *Xbp1^flx/flx^* and *AlbCre;Xbp1^flx/flx^* mice are shown. (XLSX file)

**Supplementary Data 3.** **Eigenvalue/pencil decomposition of all transcriptomes with normalized RNA-Seq quantification from the *Xbp1^flx/flx^* and *AlbCre;Xbp1^flx/flx^* mouse livers (related to Figure 2).** Period, decay rate, mathematical phase, and absolute amplitude for each gene are shown. Tab 1: Gene oscillations detected in *Xbp1^flx/flx^* mice. Tab 2: Gene oscillations detected in *AlbCre;Xbp1^flx/flx^* mice. (XLSX file)

**Supplementary Data 4.** **Rhythmicity analysis incorporating non-parametric method (RAIN) analysis of hepatic transcriptome in the *Xbp1^flx/flx^* and *AlbCre;Xbp1^flx/flx^* mice (related to Supplementary Figure 5).** Tab 1: 12-hour rhythmicity detected by RAIN in *Xbp1^flx/flx^* mice (Period set to 10-14 hours); Tab 2: 12-hour rhythmicity detected by RAIN in *AlbCre;Xbp1^flx/flx^* mice (Period set to 10-14 hours); Tab 3: 24-hour rhythmicity detected by RAIN in *Xbp1^flx/flx^* mice (Period set to 20-28 hours); Tab 4: 24-hour rhythmicity detected by RAIN in *AlbCre;Xbp1^flx/flx^* mice (Period set to 20-28 hours); Tab 5: All rhythmicity detected by RAIN in *Xbp1^flx/flx^* mice (Period set to 4-28 hours); Tab 6: All rhythmicity detected by RAIN in *AlbCre;Xbp1^flx/flx^* mice (Period set to 4-28 hours). (XLSX file)

**Supplementary Data 5.** **Normalized quantification of the targeted metabolomics from the *Xbp1^flx/flx^* and *AlbCre;Xbp1^flx/flx^* mouse livers (related to Figure 4).** Quantification results for the flow injection analysis tandem mass spectrometry (FIA-MS/MS): The total 146 analytes from the Biocrates AbsoluteIDQ p180 kit are shown. The *Xbp1^flx/flx^* and *AlbCre;Xbp1^flx/flx^* mice were fed regular chow *ad libitum* at 15- and 30-weeks of age (*n* = 4/group/age). (XLSX file)

**Supplementary Data 6.** **Quantification of the energy expenditure from the *Xbp1^flx/flx^* and *AlbCre;Xbp1^flx/flx^* mouse (related to Figure 7).** The real-time values of mouse indirect calorimetry experiments for oxygen consumption (VO2), carbon dioxide production (VCO2), energy expenditure (EE), and respiratory exchange ratio (RER) are shown with the subject mass and experiment time. The *Xbp1^flx/flx^* and *AlbCre;Xbp1^flx/flx^* mice were fed regular chow *ad libitum* at 50-60 weeks of age (*n* = 4). (XLSX file)
