# Supplementary material for: XBP1 links the 12-hour clock to NAFLD and regulation of membrane fluidity and lipid homeostasis
Source: Nat Commun. 2020 Dec 4;11:6215. doi: 10.1038/s41467-020-20028-z (PMC7718229; doi:10.1038/s41467-020-20028-z)
Supplement: Supplementary file 9 — Reporting Summary [file 41467_2020_20028_MOESM9_ESM.pdf]

nature research

Corresponding author(s): Bert W. O'Malley

Last updated by author(s): Sep 4, 2020

Reporting Summary

Nature Research wishes to improve the reproducibility of the work that we publish. This form provides structure for consistency and transparency in reporting. For further information on Nature Research policies, see Authors & Referees and the Editorial Policy Checklist .

Statistics

For all statistical analyses, confirm that the following items are present in the figure legend, table legend, main text, or Methods section.

n/a	Confirmed	
☐	☒	The exact sample size (*n*) for each experimental group/condition, given as a discrete number and unit of measurement
☐	☒	A statement on whether measurements were taken from distinct samples or whether the same sample was measured repeatedly
☐	☒	The statistical test(s) used AND whether they are one- or two-sided<br>*Only common tests should be described solely by name; describe more complex techniques in the Methods section.*
☐	☒	A description of all covariates tested
☐	☒	A description of any assumptions or corrections, such as tests of normality and adjustment for multiple comparisons
☐	☒	A full description of the statistical parameters including central tendency (e.g. means) or other basic estimates (e.g. regression coefficient) AND variation (e.g. standard deviation) or associated estimates of uncertainty (e.g. confidence intervals)
☐	☒	For null hypothesis testing, the test statistic (e.g. *F*, *t*, *r*) with confidence intervals, effect sizes, degrees of freedom and *P* value noted<br>*Give P values as exact values whenever suitable.*
☒	☐	For Bayesian analysis, information on the choice of priors and Markov chain Monte Carlo settings
☒	☐	For hierarchical and complex designs, identification of the appropriate level for tests and full reporting of outcomes
☒	☐	Estimates of effect sizes (e.g. Cohen's *d*, Pearson's *r*), indicating how they were calculated

Our web collection on statistics for biologists contains articles on many of the points above.

Software and code

Policy information about availability of computer code

Data collection	Metabolite concentrations were obtained using the AbsoluteIDQ kit p180 (Biocrates Life Science AG, Austria) according to manufacturer's instructions on an QTRAP 6500 LC/MS/MS System (AB SCIEX, USA) equipped with an electrospray ionization source, an Agilent G1367B autosampler and the Analyst 1.51 software (AB SCIEX, USA). Mito stress and fuel flex tests were performed with Seahorse kits and the Seahorse XFe96 analyzer. Immunoblot was detected by autoradiography or KwikQuant Imager (Kindle Biosciences).

Data analysis	GraphPad Prism 8 software was used to analyze the data and to make figures.<br>RNA-seq reads were aligned to the mouse genome (mm10/NCBI38) using HISAT2 (2.1.0). RNA-seq quantification was performed with htseq-count (0.9.1, default parameters) and iGenome annotation (archive-2015-07-17-14-33-26). Quantification values were normalized with DESeq2 (2.11.40.2), followed by filtering out of zero expression genes in each replicate or time point. The mean values of normalized RNA-seq data at each time point were used to determine the superimposed oscillations via the eigenvalue/pencil method as previously described (Zhu, B. et al. Cell Metab 2017; Antoulas, A. C. et al. PLoS One 2018). The same period criterion established by Hughes M. E. et al. PLoS Genet 2009 for circadian genes and 12-hour genes was used for consistency and for better comparison and understanding of the prevalence of 12-hour genes. Matlab_R2019A was used to determine the eigenvalue/pencil superimposed oscillations. RAIN package in Bioconductor (3.4) (http://www.bioconductor.org/packages/release/bioc/html/rain.html) was used per default parameters.<br>ChIP-seq sequencing reads were mapped to the mouse genome (mm10/NCBI38) with BOWTIE2 (2.3.4.2). MACS2 version 2.1.1.20160309 was used to perform the peak calling at CT0 and CT8 against their respective input for each replicate individually, and only intersecting peaks between the two biological replicates at each time point were determined as high-confidence peaks. For the CT0 to CT36 4-hour interval ChIP-seqs, pooled duplicates at each time point were used. In all the sequencing analysis, the differential sequencing reads were "down-sampled" to the lowest number of uniquely mapped reads for the normalization of sequencing depth. The ChIP-seq peak annotations and motif analysis were done by using HOMER (v4.10.3) with default parameters.<br>Gene set enrichment analysis (GSEA) with default phenotype permutation option was used for gene set enrichment against human hepatic steatosis and healthy steatosis microarray datasets obtained from a published database Ahrens, M. et al Cell Metab 2013.<br>CalR (version 1.2, https://calrapp.org) was used for indirect calorimetry to measure physiological energy balance.<br>Analytes concentrations were calculated and evaluated in the Analyst/MetIQ software. Seahorse XF Report Generator (Agilent) was used to automatically calculates the key parameters of the Seahorse XF Cell Mito Stress and Mito Fuel Flex results.

For manuscripts utilizing custom algorithms or software that are central to the research but not yet described in published literature, software must be made available to editors/reviewers. We strongly encourage code deposition in a community repository (e.g. GitHub). See the Nature Research guidelines for submitting code & software for further information.

Data

Policy information about availability of data

All manuscripts must include a data availability statement. This statement should provide the following information, where applicable:

- Accession codes, unique identifiers, or web links for publicly available datasets
- A list of figures that have associated raw data
- A description of any restrictions on data availability

The genomic datasets generated in this study can be accessed at the GEO public repository using the accession number GSE150890. The array-based mRNA expression profiling of liver samples from NAFLD and NASH patients with healthy obese and controls were obtained from GEO (GSE48325, GSE48452). The data that support the findings of this study are available in the source data files.

Field-specific reporting

Please select the one below that is the best fit for your research. If you are not sure, read the appropriate sections before making your selection.

☒ Life sciences ☐ Behavioural & social sciences ☐ Ecological, evolutionary & environmental sciences

For a reference copy of the document with all sections, see nature.com/documents/nr-reporting-summary-flat.pdf

Life sciences study design

All studies must disclose on these points even when the disclosure is negative.

Sample size	Statistical methods were not used to predetermine sample size (n). Number of sample was determined based on experimental approach, availability, feasibility required to obtain definitive results. Sample size was chosen based on our prior studies and published literature using the same types of assays to ensure statistically meaningful results. Both in vitro and in vivo studies were performed with at least three biologically independent samples per group. Statistical tests then performed using GraphPad to provide confidence in the conclusions made.
Data exclusions	No data were excluded from the analyses.
Replication	Experiments reported are replicated at least twice with similar observations.
Randomization	Animals were assigned to the experimental group based on genotype, and there were no drug treatment groups. Genetic manipulation of cells using adenovirus Cre recombinase. Therefore randomization was not utilized.
Blinding	For plasma and metabolic profiling and glucose tolerance tests of mice, actual measurements were carried out by service core members of the institute, who did not know which phenotypes were expected. Blinding was performed by removal of identifying information from each sample while primary data was collected during an experiment. Once data had been collected, blinding was not required for statistical or bioinformatic analysis as objective readouts had been used in all experiments and all samples were analyzed using an identical method for each experiment.

Reporting for specific materials, systems and methods

We require information from authors about some types of materials, experimental systems and methods used in many studies. Here, indicate whether each material, system or method listed is relevant to your study. If you are not sure if a list item applies to your research, read the appropriate section before selecting a response.

Materials & experimental systems

n/a	Involved in the study
☐	☒ Antibodies
☐	☒ Eukaryotic cell lines
☒	☐ Palaeontology
☐	☒ Animals and other organisms
☒	☐ Human research participants
☒	☐ Clinical data

Methods

n/a	Involved in the study
☐	☒ ChIP-seq
☒	☐ Flow cytometry
☒	☐ MRI-based neuroimaging

Antibodies

Antibodies used	anti-XBP1s antibody (Biolegend Poly6195, 0.5 µg per mL for immunoblot, 10 µg per 100µg of mouse liver chromatin for ChIP-Seq); anti-F4/80 (Thermo Fisher Scientific, Cat#MF48000, 1:100) and anti-Casp2 (Abcam Cat#ab2251, 1:200). Secondary antibody coupled to horseradish peroxidase (Digital anti-Rabbit-HRP, R1006, Kindle Biosciences; 1:1000).
Validation	All antibodies were validated by the supplier for human and mouse samples, and were checked in the lab by Western blotting on cell lysate, by Immunohistochemistry using mouse primary tissues, and by comparing to the manufacturer's or in-house results. See manufacturers websites for validation statements (https://www.thermofisher.com/; https:// www.abcam.com/; https:// www.biolegend.com/; https://www.kindlebio.com/) found in technical data sheets.

Eukaryotic cell lines

Policy information about cell lines

Cell line source(s)	Mouse embryonic fibroblasts (MEFs) were isolated from male Xbp1 flx/flx mice and immortalized by transfection with the SV40 Large T antigen as previously described (Zhu, B. et al. Cell Metab 2017). For Ade-Cre treatment, MEFs were infected with either adenovirus harboring an expression cassette for CRE recombinase or CRE-GFP and cultured in DMEM (4.5g/L glucose) supplemented with 10% FBS at 37 °C with 5% CO2.
Authentication	The genotypes of the cell lines were also validated by PCR (Cre) and western blotting (Xbp1 protein expression)
Mycoplasma contamination	All cell lines mentioned above undergo frequently tested for mycoplasma contamination and were verified to be mycoplasma negative before undertaking any experiments with them.
Commonly misidentified lines (See ICLAC register)	No commonly misidentified cell lines were used.

Animals and other organisms

Policy information about studies involving animals; ARRIVE guidelines recommended for reporting animal research

Laboratory animals	All animal studies were conducted in accordance with regulations of the Committee on Animal Care and Use at Baylor College of Medicine. Xbp1 flox mice were kindly provided by Dr. Xi Chen at Baylor College of Medicine, and were generated as previously described 27. Xbp1 flox mice were crossed with AlbCre transgenic mice (Jackson Laboratories) to generate AlbCre; Xbp1 flox, as well as Xbp1 flox and AlbCre littermate controls. All offspring from this cross were maintained on a C57BL/6 background. Mice were maintained on a 12h:12h light:dark cycle and allowed free access to regular chow and water under strict temperature control. This study was conducted in male mice, not female mice.
Wild animals	The study did not involve wild animals.
Field-collected samples	The study did not involve samples collected from the field.
Ethics oversight	All animal procedures were completed in accordance with the Guidelines for the Care and Use of Laboratory Animals. All animal studies were conducted in accordance with regulations of the Committee on Animal Care and Use at Baylor College of Medicine.

Note that full information on the approval of the study protocol must also be provided in the manuscript.

ChIP-seq

Data deposition

☒ Confirm that both raw and final processed data have been deposited in a public database such as GEO.

☒ Confirm that you have deposited or provided access to graph files (e.g. BED files) for the called peaks.

Data access links
May remain private before publication.

The RNA and ChIP sequencing data are deposited in the NCBI Gene Expression Omnibus (GEO; http://www.ncbi.nlm.nih.gov/geo/) with the accession number GSE150890.

Files in database submission

GSM4560206 WT_RNA-Seq_CT00_Rep1
GSM4560207 WT_RNA-Seq_CT00_Rep2
GSM4560208 WT_RNA-Seq_CT02_Rep1
GSM4560209 WT_RNA-Seq_CT02_Rep2
GSM4560210 WT_RNA-Seq_CT04_Rep1
GSM4560211 WT_RNA-Seq_CT04_Rep2
GSM4560212 WT_RNA-Seq_CT06_Rep1
GSM4560213 WT_RNA-Seq_CT06_Rep2
GSM4560214 WT_RNA-Seq_CT08_Rep1
GSM4560215 WT_RNA-Seq_CT08_Rep2
GSM4560216 WT_RNA-Seq_CT10_Rep1
GSM4560217 WT_RNA-Seq_CT10_Rep2
GSM4560218 WT_RNA-Seq_CT12_Rep1
GSM4560219 WT_RNA-Seq_CT12_Rep2
GSM4560220 WT_RNA-Seq_CT14_Rep1
GSM4560221 WT_RNA-Seq_CT14_Rep2
GSM4560222 WT_RNA-Seq_CT16_Rep1
GSM4560223 WT_RNA-Seq_CT16_Rep2
GSM4560224 WT_RNA-Seq_CT18_Rep1
GSM4560225 WT_RNA-Seq_CT18_Rep2
GSM4560226 WT_RNA-Seq_CT20_Rep1
GSM4560227 WT_RNA-Seq_CT20_Rep2
GSM4560228 WT_RNA-Seq_CT22_Rep1
GSM4560229 WT_RNA-Seq_CT22_Rep2
GSM4560230 WT_RNA-Seq_CT24_Rep1
GSM4560231 WT_RNA-Seq_CT24_Rep2
GSM4560232 WT_RNA-Seq_CT26_Rep1
GSM4560233 WT_RNA-Seq_CT26_Rep2
GSM4560234 WT_RNA-Seq_CT28_Rep1
GSM4560235 WT_RNA-Seq_CT28_Rep2
GSM4560236 WT_RNA-Seq_CT30_Rep1
GSM4560237 WT_RNA-Seq_CT30_Rep2
GSM4560238 WT_RNA-Seq_CT32_Rep1
GSM4560239 WT_RNA-Seq_CT32_Rep2
GSM4560240 WT_RNA-Seq_CT34_Rep1
GSM4560241 WT_RNA-Seq_CT34_Rep2
GSM4560242 WT_RNA-Seq_CT36_Rep1
GSM4560243 WT_RNA-Seq_CT36_Rep2
GSM4560244 WT_RNA-Seq_CT38_Rep1
GSM4560245 WT_RNA-Seq_CT38_Rep2
GSM4560246 WT_RNA-Seq_CT40_Rep1
GSM4560247 WT_RNA-Seq_CT40_Rep2
GSM4560248 WT_RNA-Seq_CT42_Rep1
GSM4560249 WT_RNA-Seq_CT42_Rep2
GSM4560250 WT_RNA-Seq_CT44_Rep1
GSM4560251 WT_RNA-Seq_CT44_Rep2
GSM4560252 WT_RNA-Seq_CT46_Rep1
GSM4560253 WT_RNA-Seq_CT46_Rep2
GSM4560254 XBP1_LKO_RNA-Seq_CT00_Rep1
GSM4560255 XBP1_LKO_RNA-Seq_CT00_Rep2
GSM4560256 XBP1_LKO_RNA-Seq_CT02_Rep1
GSM4560257 XBP1_LKO_RNA-Seq_CT02_Rep2
GSM4560258 XBP1_LKO_RNA-Seq_CT04_Rep1
GSM4560259 XBP1_LKO_RNA-Seq_CT04_Rep2
GSM4560260 XBP1_LKO_RNA-Seq_CT06_Rep1
GSM4560261 XBP1_LKO_RNA-Seq_CT06_Rep2
GSM4560262 XBP1_LKO_RNA-Seq_CT08_Rep1
GSM4560263 XBP1_LKO_RNA-Seq_CT08_Rep2
GSM4560264 XBP1_LKO_RNA-Seq_CT10_Rep1
GSM4560265 XBP1_LKO_RNA-Seq_CT10_Rep2
GSM4560266 XBP1_LKO_RNA-Seq_CT12_Rep1
GSM4560267 XBP1_LKO_RNA-Seq_CT12_Rep2
GSM4560268 XBP1_LKO_RNA-Seq_CT14_Rep1
GSM4560269 XBP1_LKO_RNA-Seq_CT14_Rep2

GSM4560270 XBP1_LKO_RNA-Seq_CT16_Rep1
GSM4560271 XBP1_LKO_RNA-Seq_CT16_Rep2
GSM4560272 XBP1_LKO_RNA-Seq_CT18_Rep1
GSM4560273 XBP1_LKO_RNA-Seq_CT18_Rep2
GSM4560274 XBP1_LKO_RNA-Seq_CT20_Rep1
GSM4560275 XBP1_LKO_RNA-Seq_CT20_Rep2
GSM4560276 XBP1_LKO_RNA-Seq_CT22_Rep1
GSM4560277 XBP1_LKO_RNA-Seq_CT22_Rep2
GSM4560278 XBP1_LKO_RNA-Seq_CT24_Rep1
GSM4560279 XBP1_LKO_RNA-Seq_CT24_Rep2
GSM4560280 XBP1_LKO_RNA-Seq_CT26_Rep1
GSM4560281 XBP1_LKO_RNA-Seq_CT26_Rep2
GSM4560282 XBP1_LKO_RNA-Seq_CT28_Rep1
GSM4560283 XBP1_LKO_RNA-Seq_CT28_Rep2
GSM4560284 XBP1_LKO_RNA-Seq_CT30_Rep1
GSM4560285 XBP1_LKO_RNA-Seq_CT30_Rep2
GSM4560286 XBP1_LKO_RNA-Seq_CT32_Rep1
GSM4560287 XBP1_LKO_RNA-Seq_CT32_Rep2
GSM4560288 XBP1_LKO_RNA-Seq_CT34_Rep1
GSM4560289 XBP1_LKO_RNA-Seq_CT34_Rep2
GSM4560290 XBP1_LKO_RNA-Seq_CT36_Rep1
GSM4560291 XBP1_LKO_RNA-Seq_CT36_Rep2
GSM4560292 XBP1_LKO_RNA-Seq_CT38_Rep1
GSM4560293 XBP1_LKO_RNA-Seq_CT38_Rep2
GSM4560294 XBP1_LKO_RNA-Seq_CT40_Rep1
GSM4560295 XBP1_LKO_RNA-Seq_CT40_Rep2
GSM4560296 XBP1_LKO_RNA-Seq_CT42_Rep1
GSM4560297 XBP1_LKO_RNA-Seq_CT42_Rep2
GSM4560298 XBP1_LKO_RNA-Seq_CT44_Rep1
GSM4560299 XBP1_LKO_RNA-Seq_CT44_Rep2
GSM4560300 XBP1_LKO_RNA-Seq_CT46_Rep1
GSM4560301 XBP1_LKO_RNA-Seq_CT46_Rep2
GSM4560302 XBP1s_ChIP_CT00
GSM4560303 XBP1s_ChIP_CT04
GSM4560304 XBP1s_ChIP_CT08
GSM4560305 XBP1s_ChIP_CT12
GSM4560306 XBP1s_ChIP_CT16
GSM4560307 XBP1s_ChIP_CT20
GSM4560308 XBP1s_ChIP_CT24
GSM4560309 XBP1s_ChIP_CT28
GSM4560310 XBP1s_ChIP_CT32
GSM4560311 XBP1s_ChIP_CT36
GSM4560312 XBP1s_ChIP_LKO
GSM4560313 XBP1_WT_CT0_ChIP_seq_Input
GSM4560314 XBP1_WT_CT0_ChIP_seq_XBP1sChIP
GSM4560315 XBP1_WT_CT8_ChIP_seq_Input
GSM4560316 XBP1_WT_CT8_ChIP_seq_XBP1sChIP
GSM4560317 XBP1_LKO_ChIP_seq_Input
GSM4560318 XBP1_LKO_ChIP_seq_XBP1sChIP

Genome browser session
(e.g. UCSC)

no longer applicable

Methodology

Replicates

two biological replicates of mouse liver tissues were used per genotype/time point for the The RNA and ChIP sequencing

Sequencing depth

an average of 40 million paired-end reads per sample for RNA sequencing. For ChIP sequencing, the comprehensive CT0 and CT8 DNA libraries generated ~40 million 75 bp paired-end reads per sample; and the CT0 to CT36 time point DNA libraries generated ~30 million 75 bp single-end reads per sample.

Antibodies

anti-XBP1s antibody (Biolegend Poly6195) for ChIP sequencing.

Peak calling parameters

Narrow peaks were called using the callpeak function from MACS2 v2.1.1.20160309.4 against matched input samples, using default parameters and a q-value cutoff of 0.05, according to the ENCODE transcription factor ChIP-seq Data Standards and Processing Pipeline (https://www.encodeproject.org/chip-seq/transcription_factor/).

Data quality	In all the sequencing analysis, the differential sequencing reads were "down-sampled" to the lowest number of uniquely mapped reads for the normalization of sequencing depth, and only intersecting peaks between the two biological replicates at each time point were determined as high-confidence peaks.
Software	ChIP-seq sequencing reads were mapped to the mouse genome (mm10/NCBI38) with BOWTIE2 (2.3.4.2). MACS2 version 2.1.1.20160309 was used to perform the peak calling against their respective input for each replicate individually.
