# Supplementary material for: XBP1 links the 12-hour clock to NAFLD and regulation of membrane fluidity and lipid homeostasis
Source: Nat Commun. 2020 Dec 4;11:6215. doi: 10.1038/s41467-020-20028-z (PMC7718229; doi:10.1038/s41467-020-20028-z)

Uncropped gel pictures
Supplementary Fig. 1b

XBP1s WB

~55-60 kDa

~70 kDa -
~55 kDa -
~35 kDa -
~25 kDa -
~15 kDa -

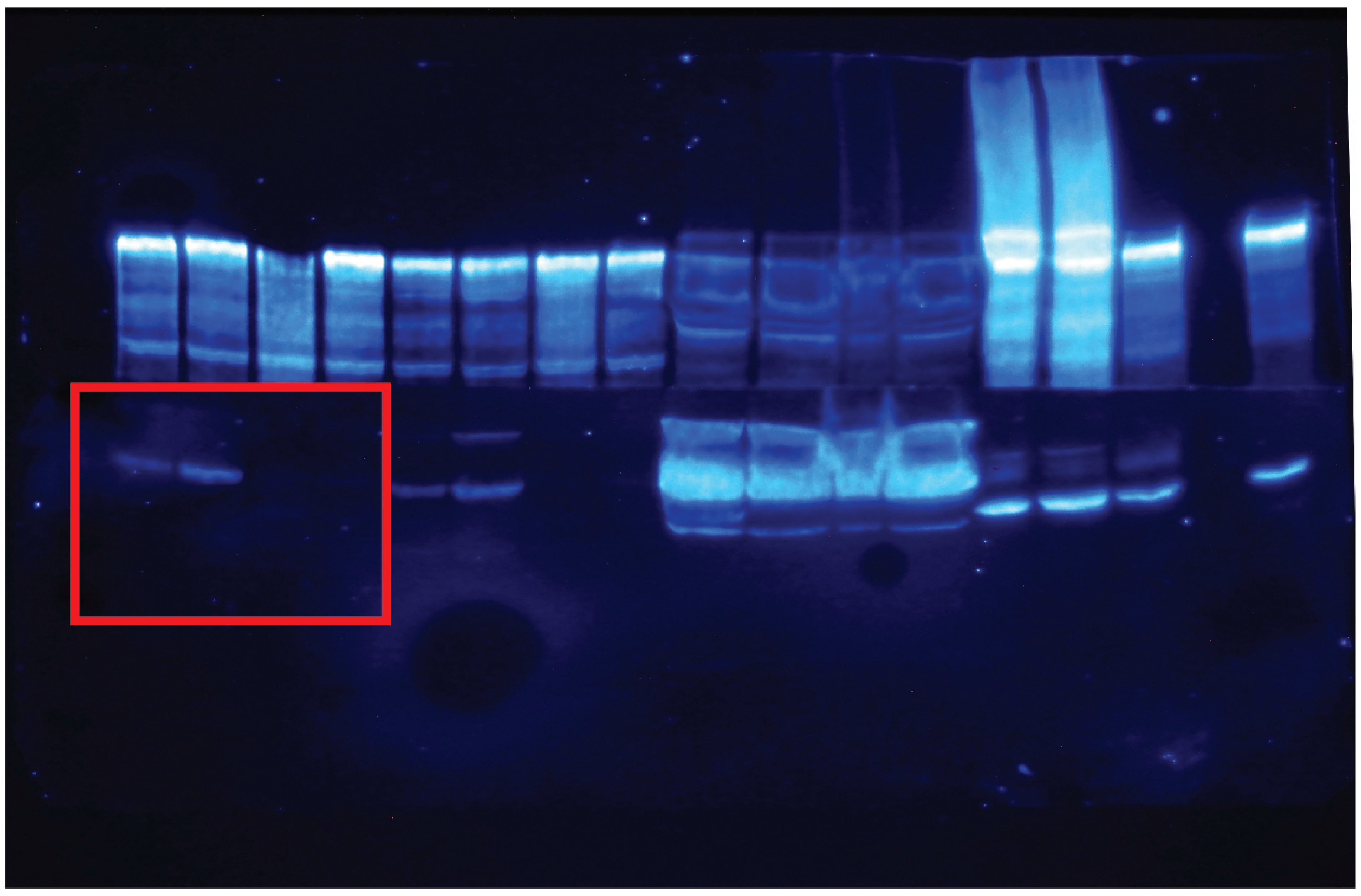

Upper gel:
cropped for another unrelated antibody

← cropped line before primary antibody incubation

~70 kDa -
~55 kDa -
~35 kDa -
~25 kDa -
~15 kDa -

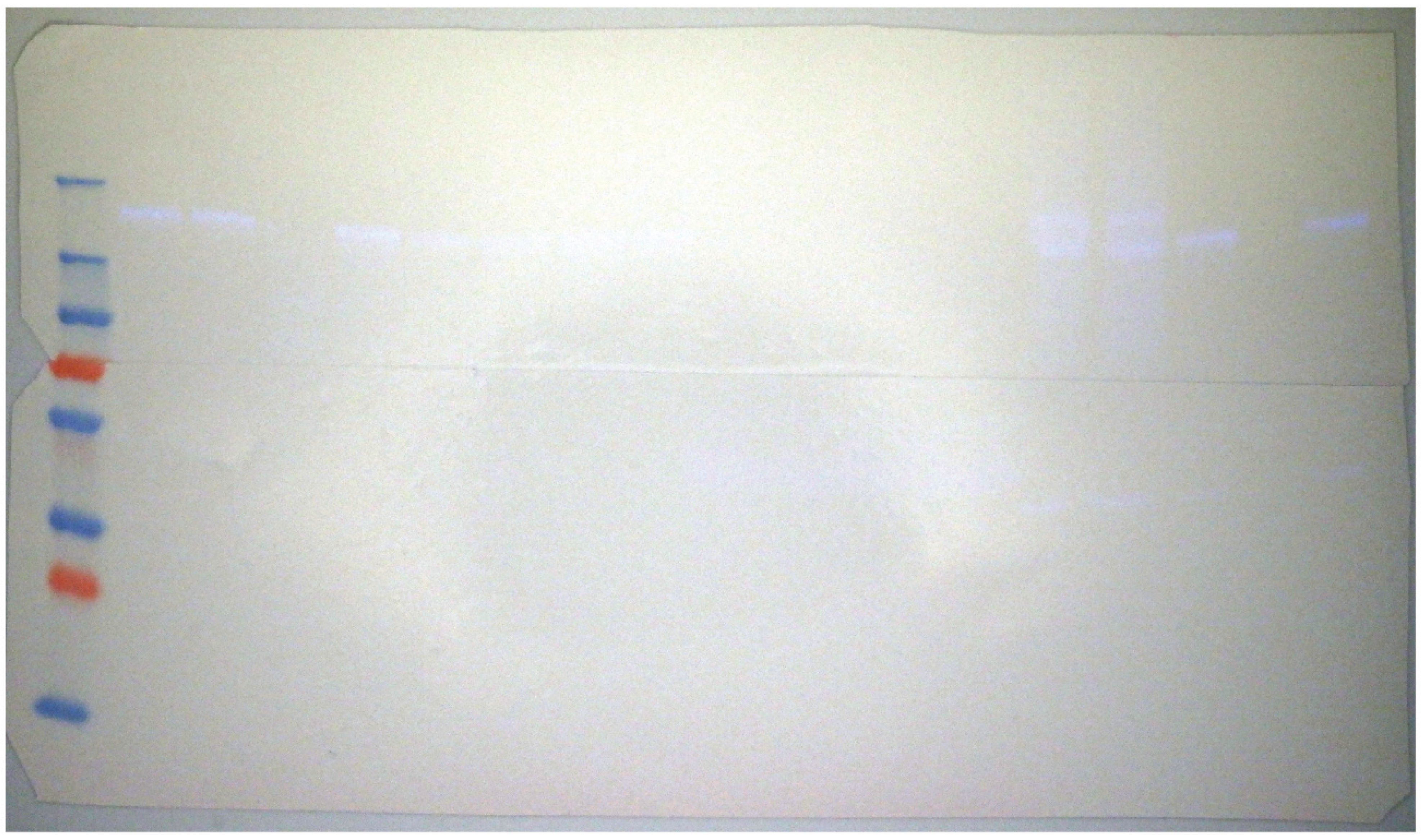

Upper gel:
cropped for another unrelated antibody

← cropped line before primary antibody incubation

Ponceau Staining

~70 kDa -
~55 kDa -
~35 kDa -
~25 kDa -
~15 kDa -

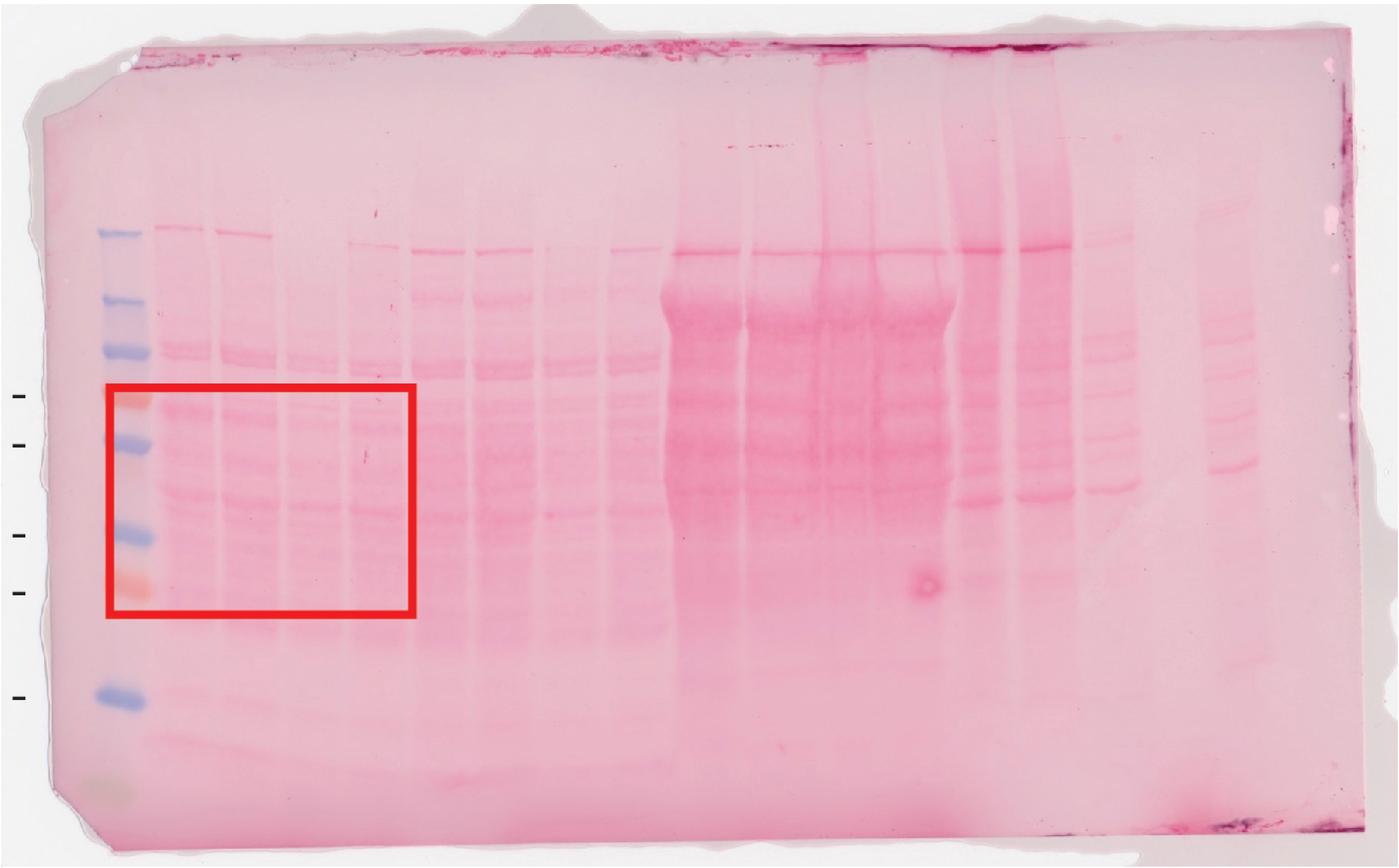

Supplement: Supplementary file 10 — Source Data [file 41467_2020_20028_MOESM10_ESM.zip › Source Data/Source Data Support Supplementary Figure 1.pdf]
