# Supplementary material for: XBP1 links the 12-hour clock to NAFLD and regulation of membrane fluidity and lipid homeostasis
Source: Nat Commun. 2020 Dec 4;11:6215. doi: 10.1038/s41467-020-20028-z (PMC7718229; doi:10.1038/s41467-020-20028-z)
Supplement: Supplementary file 10 — Source Data [file 41467_2020_20028_MOESM10_ESM.zip › Source Data/Source Data Support Supplementary Figure 12.pdf]

GLM

Effect	Full Day Mass	Full Day Group	Full Day Interaction	Light Mass	Light Group	Light Interaction	Dark Mass	Dark Group	Dark Interaction
Hourly Food Consumed (kcal)	0.3081	0.6215		0.3249	0.9204		0.6696	0.5350	
Total Food Consumed (kcal)	0.7125	0.2735		0.8351	0.2531		0.6228	0.2907	
Oxygen Consumption (ml/hr)	0.4824	0.8463		0.2846	0.8692		0.7853	0.8256	
Carbon Dioxide Production (ml/hr)	0.4972	0.6103		0.3434	0.8751		0.6808	0.4268	
Energy Expenditure (kcal/hour)	0.2942	0.0281 *		0.8082	0.0319 *		0.0873	0.0454 *	
Energy Balance (kcal/hour)	0.4075	0.4233		0.3124	0.8579		0.8223	0.4388	

ANOVA

Effect	Full Day Group	Light Group	Dark Group
Respiratory Exchange Ratio	0.2647	0.9305	0.0301 *
Locomotor Activity (beam breaks)	0.9184	0.8685	0.9800
Ambulatory Activity (beam breaks)	0.7469	0.3205	0.8616

Mass effect: Lean.Mass

Signif. codes: <0.001 ***,<0.01 **,<0.05 *
