# Supplementary material for: XBP1 links the 12-hour clock to NAFLD and regulation of membrane fluidity and lipid homeostasis
Source: Nat Commun. 2020 Dec 4;11:6215. doi: 10.1038/s41467-020-20028-z (PMC7718229; doi:10.1038/s41467-020-20028-z)
Supplement: Supplementary file 10 — Source Data [file Source Data Support Supplementary Figure 10 high resolution.pdf › Source Data/Source Data Support Supplementary Figure 10 high resolution.pdf]

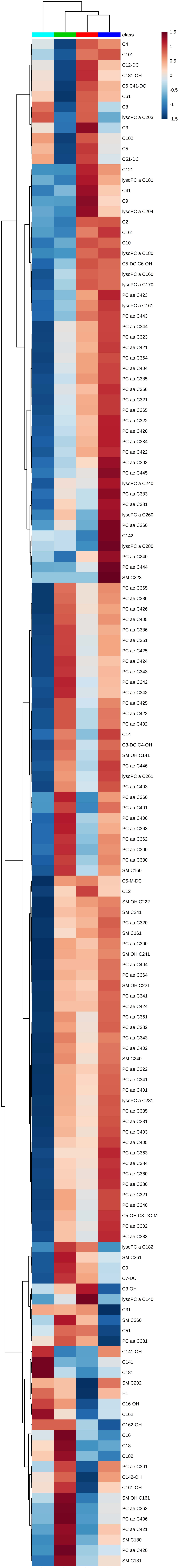
class
1.5
1
0.5
0
-0.5
-1
-1.5
C4
C101
C12-DC
C181-OH
C6 C41-DC
C61
C8
lysoPC a C203
C3
C102
C5
C51-DC
C121
lysoPC a C181
C41
C9
lysoPC a C204
C2
C161
C10
lysoPC a C180
C5-DC C6-OH
lysoPC a C160
lysoPC a C170
PC ae C423
lysoPC a C161
PC ae C443
PC aa C344
PC aa C323
PC aa C421
PC aa C364
PC ae C404
PC aa C385
PC aa C366
PC aa C321
PC aa C365
PC aa C322
PC ae C420
PC aa C384
PC ae C422
PC aa C302
PC ae C445
lysoPC a C240
PC aa C383
PC aa C381
lysoPC a C260
PC aa C260
C142
lysoPC a C280
PC aa C240
PC ae C444
SM C223
PC ae C365
PC ae C386
PC aa C426
PC ae C405
PC aa C386
PC aa C361
PC ae C425
PC aa C424
PC aa C343
PC aa C342
PC ae C342
PC aa C425
PC aa C422
PC aa C402
C14
C3-DC C4-OH
SM OH C141
PC ae C446
lysoPC a C261
PC aa C403
PC aa C360
PC aa C401
PC aa C406
PC aa C363
PC aa C362
PC aa C300
PC aa C380
SM C160
C5-M-DC
C12
SM OH C222
SM C241
PC aa C320
SM C161
PC ae C300
SM OH C241
PC ae C404
PC ae C364
SM OH C221
PC aa C341
PC ae C424
PC ae C361
PC ae C382
PC ae C343
PC ae C402
SM C240
PC ae C322
PC ae C341
PC ae C401
lysoPC a C281
PC ae C385
PC aa C281
PC ae C403
PC ae C405
PC ae C363
PC ae C384
PC ae C360
PC ae C380
PC ae C321
PC ae C340
C5-OH C3-DC-M
PC ae C302
PC ae C383
lysoPC a C182
SM C261
C0
C7-DC
C3-OH
lysoPC a C140
C31
SM C260
C51
PC ae C381
C141-OH
C141
C181
SM C202
H1
C16-OH
C162
C162-OH
C16
C18
C182
PC ae C301
C142-OH
C161-OH
SM OH C161
PC ae C362
PC aa C406
PC aa C421
SM C180
PC aa C420
SM C181
