# Supplementary material for: XBP1 links the 12-hour clock to NAFLD and regulation of membrane fluidity and lipid homeostasis
Source: Nat Commun. 2020 Dec 4;11:6215. doi: 10.1038/s41467-020-20028-z (PMC7718229; doi:10.1038/s41467-020-20028-z)
Supplement: Supplementary file 10 — Source Data [file Source Data Support Supplementary Figure 3.pdf › Source Data/Source Data Support Supplementary Figure 3.pdf]

a

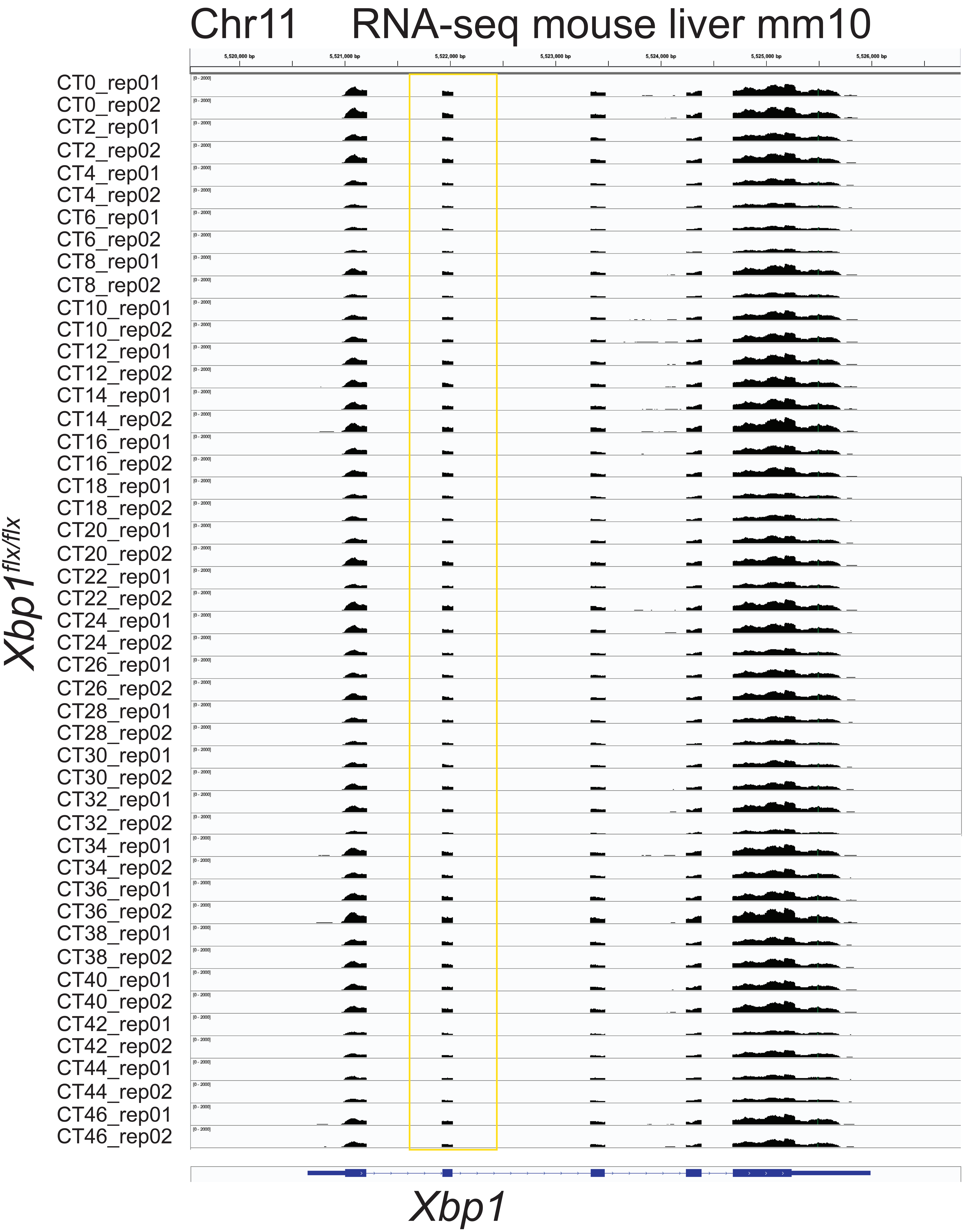
Chr11
RNA-seq mouse liver mm10
5,520,000 bp
5,521,000 bp
5,522,000 bp
5,523,000 bp
5,524,000 bp
5,525,000 bp
5,526,000 bp
CT0_rep01
CT0_rep02
CT2_rep01
CT2_rep02
CT4_rep01
CT4_rep02
CT6_rep01
CT6_rep02
CT8_rep01
CT8_rep02
CT10_rep01
CT10_rep02
CT12_rep01
CT12_rep02
CT14_rep01
CT14_rep02
CT16_rep01
CT16_rep02
CT18_rep01
CT18_rep02
CT20_rep01
CT20_rep02
CT22_rep01
CT22_rep02
CT24_rep01
CT24_rep02
CT26_rep01
CT26_rep02
CT28_rep01
CT28_rep02
CT30_rep01
CT30_rep02
CT32_rep01
CT32_rep02
CT34_rep01
CT34_rep02
CT36_rep01
CT36_rep02
CT38_rep01
CT38_rep02
CT40_rep01
CT40_rep02
CT42_rep01
CT42_rep02
CT44_rep01
CT44_rep02
CT46_rep01
CT46_rep02
Xbp1flx/flx
Xbp1

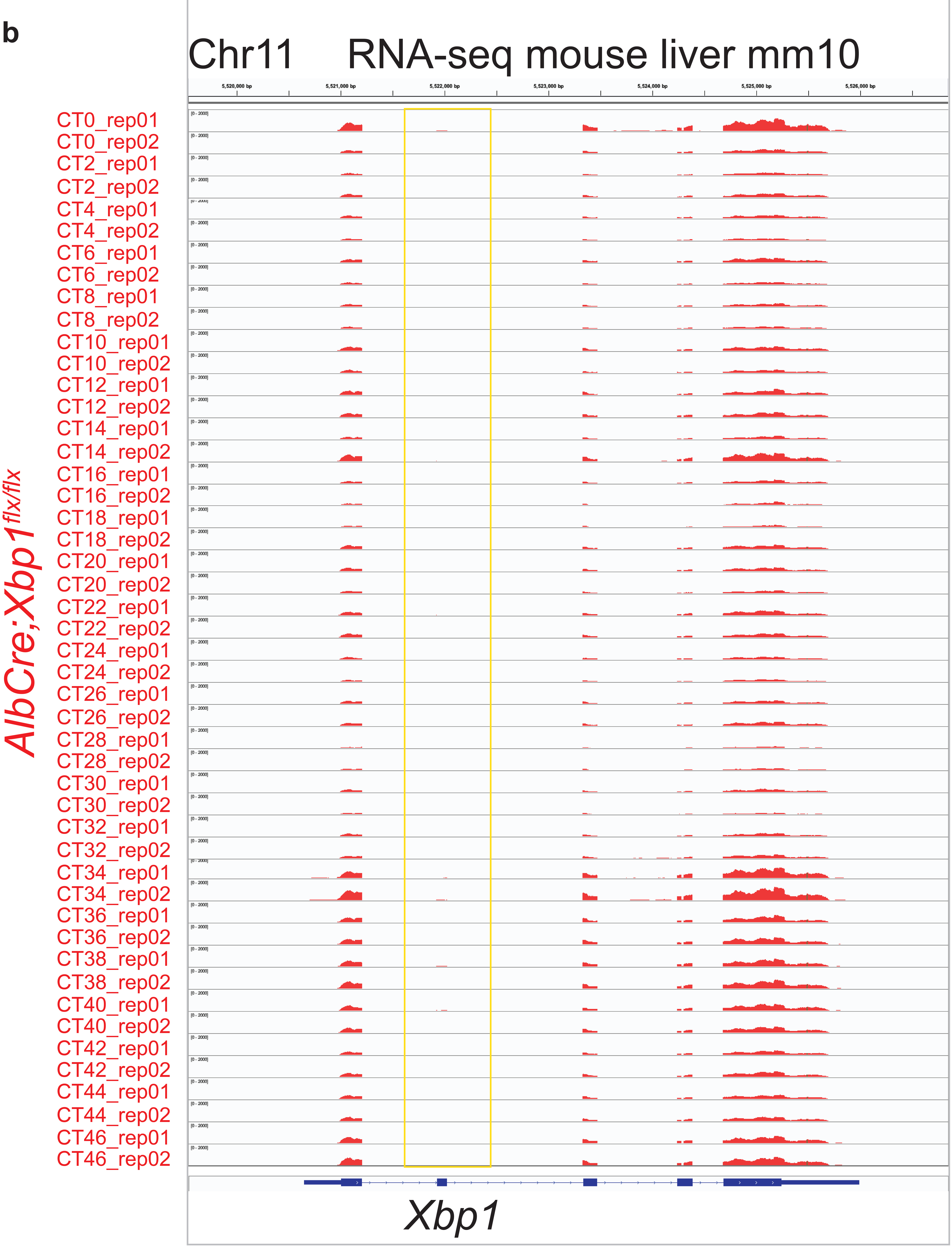

b
Chr11 RNA-seq mouse liver mm10
5,520,000 bp
5,521,000 bp
5,522,000 bp
5,523,000 bp
5,524,000 bp
5,525,000 bp
5,526,000 bp
AlbCre;Xbp1flx/flx
CT0_rep01
CT0_rep02
CT2_rep01
CT2_rep02
CT4_rep01
CT4_rep02
CT6_rep01
CT6_rep02
CT8_rep01
CT8_rep02
CT10_rep01
CT10_rep02
CT12_rep01
CT12_rep02
CT14_rep01
CT14_rep02
CT16_rep01
CT16_rep02
CT18_rep01
CT18_rep02
CT20_rep01
CT20_rep02
CT22_rep01
CT22_rep02
CT24_rep01
CT24_rep02
CT26_rep01
CT26_rep02
CT28_rep01
CT28_rep02
CT30_rep01
CT30_rep02
CT32_rep01
CT32_rep02
CT34_rep01
CT34_rep02
CT36_rep01
CT36_rep02
CT38_rep01
CT38_rep02
CT40_rep01
CT40_rep02
CT42_rep01
CT42_rep02
CT44_rep01
CT44_rep02
CT46_rep01
CT46_rep02
Xbp1
