# Supplementary material for: XBP1 links the 12-hour clock to NAFLD and regulation of membrane fluidity and lipid homeostasis
Source: Nat Commun. 2020 Dec 4;11:6215. doi: 10.1038/s41467-020-20028-z (PMC7718229; doi:10.1038/s41467-020-20028-z)
Supplement: Supplementary file 10 — Source Data [file Source Data Support Supplementary Figure 9 high resolution.pdf › Source Data/Source Data Support Supplementary Figure 9 high resolution.pdf]

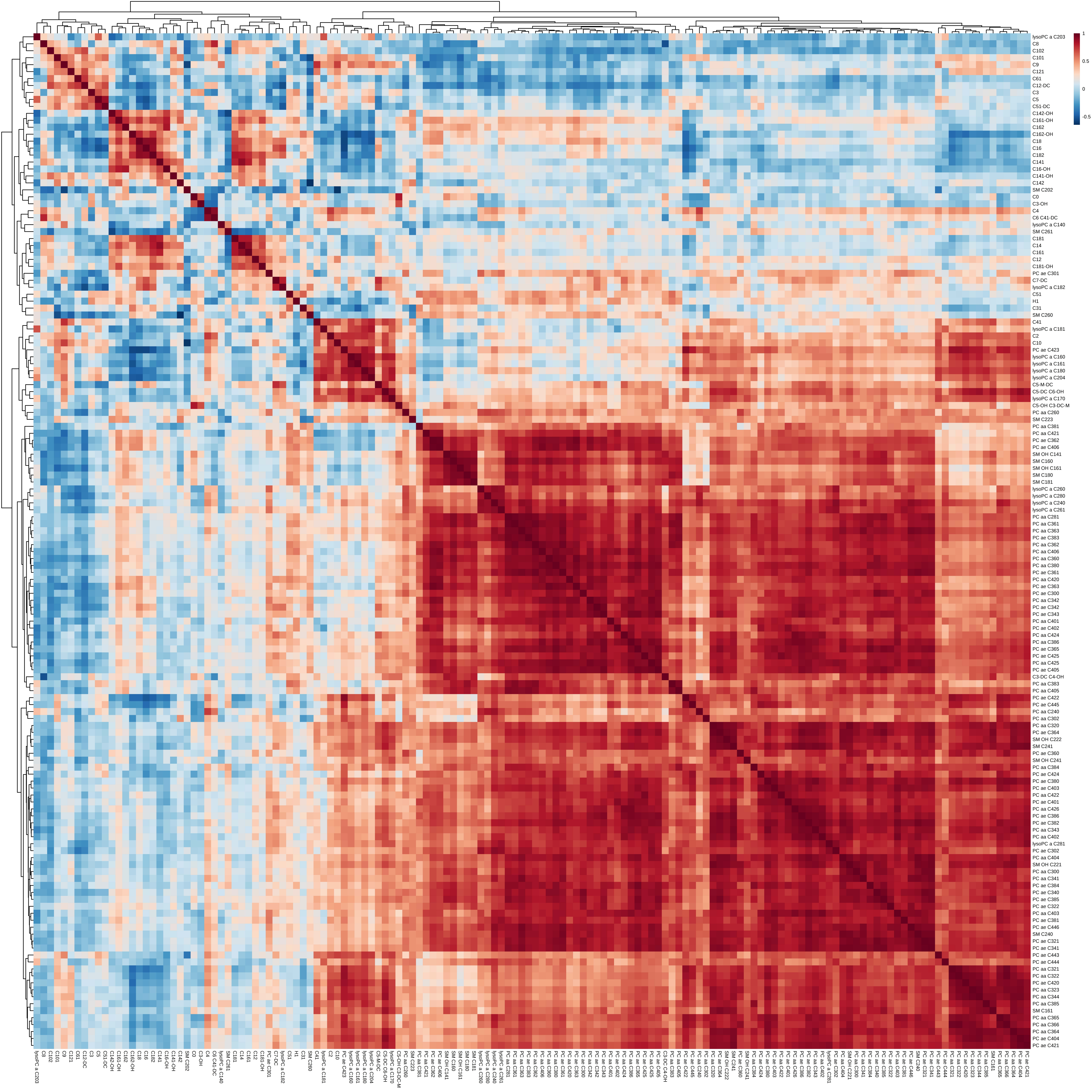
lysoPC a C203
C8
C102
C101
C9
C121
C61
C12-DC
C3
C5
C51-DC
C142-OH
C161-OH
C162
C162-OH
C18
C16
C182
C141
C16-OH
C141-OH
C142
SM C202
C0
C3-OH
C4
C6 C41-DC
lysoPC a C140
SM C261
C181
C14
C161
C12
C181-OH
PC ae C301
C7-DC
lysoPC a C182
C51
H1
C31
SM C260
C41
lysoPC a C181
C2
C10
PC ae C423
lysoPC a C160
lysoPC a C161
lysoPC a C180
lysoPC a C204
C5-M-DC
C5-DC C6-OH
lysoPC a C170
C5-OH C3-DC-M
PC aa C260
SM C223
PC aa C381
PC aa C421
PC ae C362
PC ae C406
SM OH C141
SM C160
SM OH C161
SM C180
SM C181
lysoPC a C260
lysoPC a C280
lysoPC a C240
lysoPC a C261
PC aa C281
PC aa C361
PC aa C363
PC ae C383
PC aa C362
PC aa C406
PC aa C360
PC aa C380
PC ae C361
PC aa C420
PC ae C363
PC ae C300
PC ae C342
PC aa C342
PC aa C343
PC aa C401
PC aa C402
PC aa C424
PC aa C386
PC ae C365
PC ae C425
PC aa C425
PC ae C405
C3-DC C4-OH
PC aa C383
PC aa C405
PC ae C422
PC ae C445
PC aa C240
PC aa C302
PC aa C320
PC ae C364
SM OH C222
SM C241
PC ae C360
SM OH C241
PC aa C384
PC ae C424
PC ae C380
PC ae C403
PC aa C422
PC ae C401
PC aa C426
PC ae C386
PC aa C382
PC ae C343
PC ae C402
lysoPC a C281
PC ae C302
PC aa C404
SM OH C221
PC aa C300
PC aa C341
PC ae C384
PC ae C340
PC ae C385
PC ae C322
PC aa C403
PC aa C381
PC ae C446
SM C240
PC ae C321
PC ae C341
PC ae C443
PC ae C444
PC aa C321
PC aa C322
PC ae C420
PC aa C323
PC ae C344
PC aa C385
SM C161
PC aa C365
PC aa C366
PC aa C364
PC ae C404
PC ae C421
1
0.5
0
-0.5
